# Nature’s Palette: Characterization of Shared Pigments in Colorful Avian and Mollusk Shells

**DOI:** 10.1371/journal.pone.0143545

**Published:** 2015-12-09

**Authors:** Aida Verdes, Wooyoung Cho, Marouf Hossain, Patricia L. R. Brennan, Daniel Hanley, Tomáš Grim, Mark E. Hauber, Mandë Holford

**Affiliations:** 1 The Graduate Center, City University of New York, New York, New York, United States of America; 2 Division of Invertebrate Zoology, American Museum of Natural History, New York, New York, United States of America; 3 Department of Chemistry, Hunter College Belfer Research Building, City University of New York, New York, New York, United States of America; 4 Department of Psychology, University of Massachusetts, Amherst, Massachusetts, United States of America; 5 Department of Zoology and Laboratory of Ornithology, Palacký University, Olomouc, Czech Republic; 6 Department of Psychology, Hunter College, City University of New York, New York, New York, United States of America; University of California, UNITED STATES

## Abstract

Pigment-based coloration is a common trait found in a variety of organisms across the tree of life. For example, calcareous avian eggs are natural structures that vary greatly in color, yet just a handful of tetrapyrrole pigment compounds are responsible for generating this myriad of colors. To fully understand the diversity and constraints shaping nature’s palette, it is imperative to characterize the similarities and differences in the types of compounds involved in color production across diverse lineages. Pigment composition was investigated in eggshells of eleven paleognath bird taxa, covering several extinct and extant lineages, and shells of four extant species of mollusks. Birds and mollusks are two distantly related, calcareous shell-building groups, thus characterization of pigments in their calcareous structures would provide insights to whether similar compounds are found in different phyla (Chordata and Mollusca). An ethylenediaminetetraacetic acid (EDTA) extraction protocol was used to analyze the presence and concentration of biliverdin and protoporphyrin, two known and ubiquitous tetrapyrrole avian eggshell pigments, in all avian and molluscan samples. Biliverdin was solely detected in birds, including the colorful eggshells of four tinamou species. In contrast, protoporphyrin was detected in both the eggshells of several avian species and in the shells of all mollusks. These findings support previous hypotheses about the ubiquitous deposition of tetrapyrroles in the eggshells of various bird lineages and provide evidence for its presence also across distantly related animal taxa.

## Introduction

Evolutionary processes acting on color production mechanisms have generated an astounding diversity of colors and patterns in animals that serve many communicative functions, including parasitic mimicry, antipredator defenses, prey-attraction, sexual displays, parent-offspring communication and other intraspecific mechanisms [1,2]. Pigments can also contribute to other functions such as thermoregulation, photoprotection, structural support, and microbial resistance [3,4].

The chemical basis and the evolutionary trajectories of pigmentation and the resulting color patterns in animals have been studied intensively [1–3]. The enormous variety of colors displayed by animals is generally determined by just a handful of low molecular weight pigment compounds, including chromoproteins that require a small non-peptide molecule or metal ion prosthetic group [5]. Animal coloration can also be the result of the specific arrangement of tissues and pigments at a nanostructural scale (i.e. structural coloration) [6–8] or chemical reactions such as bioluminescence [9], that maximize the potential coverage of these biological tissues within perceptual color spaces [10].

**Fig 1:**
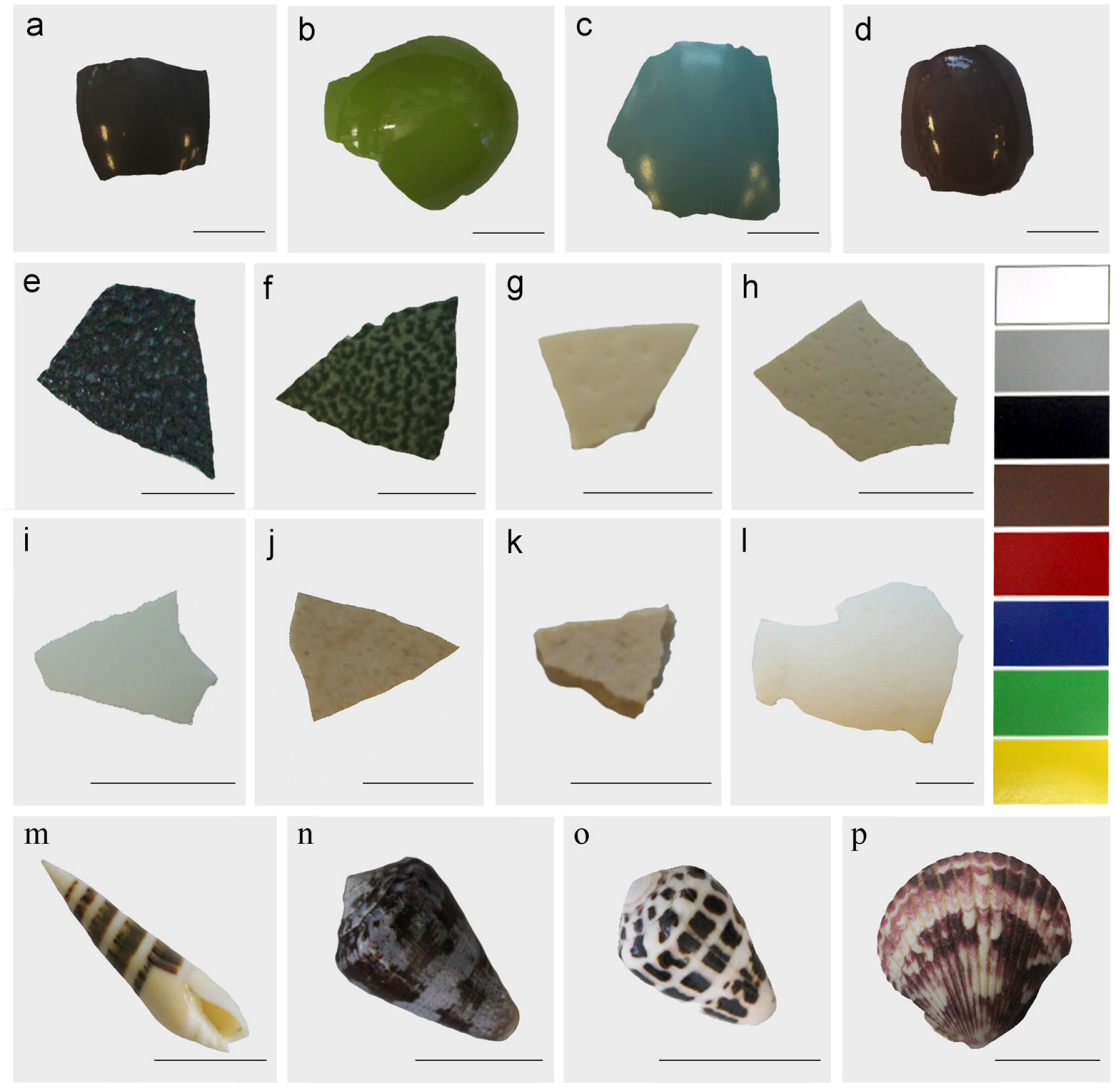
Avian eggshell fragments and molluscan shells analyzed. **(a)** *Nothoprocta perdicaria* **(b)** *Eudromia elegans* **(c)** *Tinamus major* **(d)** *Nothura maculosa* **(e)** *Dromaius novaehollandiae* **(f)** *Casuarius casuarius* **(g)** *Struthio camelus* **(h)** *Rhea americana* **(i)** *Apteryx mantelli* **(j)** *Euryapteryx curtus* **(k)** *Aepyornis* sp. **(l)** *Alligator mississippiensis* **(m)** *Hastula hectica* **(n)** *Conus purpurascens* **(o)** *Conus ebraeus* **(p)** *Argopecten* sp. Color sample card shown as color standard, sourced from Home Depot, Reno, NV (USA). Black scale bar 1 cm.

To better understand the evolutionary diversity and functions of natural colors, it is imperative to fully characterize the components and mechanisms of color production. In turn, to delimit the biological constraints acting on the evolutionary diversity of coloration, it is also crucial to examine cases of functional convergence, through redundancy or degeneracy, where similar traits and mechanisms evolved independently in unrelated lineages, and are present in analogous forms or functions in different taxa [4,11]. Here we provide comparative data on the pigmentation and coloration of calcareous external structures produced by two, distantly related, lineages: birds and mollusks (Fig 1). Specifically, we examine the chemical basis of avian eggshell and mollusk shell colors and identify whether the same shared pigments are used universally across these different taxa.

Animal pigments generate diverse colors, and their chemical bases include carotenoids, melanin, pteridines, psittacofulvins and tetrapyrroles, among others [12]. Regardless of vast evolutionary and ecological differences, bird eggs and mollusk shells share a similar diversity in coloration (at least to the human eye), and in both taxa pigments are embedded within a calcium carbonate shell matrix [13–15]. For these reasons we expect that pigment convergence may be found in these groups.

Despite a relatively simple palette of pigment compounds, birds' eggs display a wide range of colors [16]. All the variation in the coloration of birds' eggshells is thus far known to be the product of just two ancient and highly conserved [17], tetrapyrrole pigments: biliverdin IXα, and protoporphyrin IX [18–21]. While the genetic and biochemical mechanisms by which colorful eggshell pigments are formed are not yet fully understood [22], the prevailing consensus is that protoporphyrin, a cyclic tetrapyrrole which produces red-brown maculation [23], is an immediate precursor of the heme molecule, whereas biliverdin, a linear tetrapyrrole which gives the eggshell a blue-green hue, is a byproduct of hemoglobin breakdown [18–20,24]. The basic biochemistry of avian eggshell pigments has received considerable attention [17,19–21] and subsequently, the presence or absence of protoporphyrin and biliverdin in eggshells has been documented for several extinct moa species [17] and more than 100 species of extant birds [18]. The relatively well known biochemistry of avian eggshell pigments has allowed to link the coloration and patterning in birds eggs to many functions, within and beyond the two categories of communication and thermoregulation [25], including crypsis to avoid predation [26,27], increasing eggshell strength [28], providing protection from solar radiation [29], preventing overheating of embryos [30], brood parasite egg mimicry, or signaling between conspecifics [26,31].

In contrast to avian eggshells [17,19–21], there is relatively little research focused on the chemical make-up of molluscan shell pigmentation [13,32,33], despite the high biodiversity of this group [34]. Only a handful of studies have explicitly linked identified pigments to snail shell color [13]. Reports on molluscan pigments in literature are limited to a variety of unidentified small molecular compounds or pyrrole-based pigments such as porphyrin (mainly uroporphyrin), detected in several marine mollusks, including scaphopods, gastropods and bivalves. Some studies have also identified blue-green and red bile pigments and hypothesized they correspond to biliverdin [35] and biladiene [36] respectively. However, none of these pigments have been fully characterized or quantified due to methodological limitations in these early pioneering studies [32,37,38]. More recently, some studies [13,39] have identified polyene pigments in colored parts of the shells of cephalopods, bivalves and gastropods, and suggested that the color of a given molluscan shell is due to several compounds, at least one usually being a polyene [13].

Despite the scarce data on molluscan pigments, shell coloration in mollusks has been proposed to subserve different functions, such as communication and thermoregulation [40]. In some molluscan species, cryptic color and morphology are correlated with a particular habitat patch and consequently those individuals have better survival rates due to predator avoidance [38]. However, many molluscan species hide their colored surfaces in the substrate, beneath an opaque outer layer (periostracum), or under epibiont organisms, which precludes the use of these traits as cues or signals in many situations [41]. Similar to avian eggshell pigmentation, identifying the pigments responsible for molluscan shell coloration can shed light on both the biological functions and the evolutionary origins of shell pigmentation in mollusks. Here we focused on the two well-studied avian eggshell tetrapyrrole pigments: biliverdin and protoporphyrin [19], and investigated their presence in the less studied pigmentation of marine mollusk shells.

In the molluscan literature, a variety of functions and selection pressures have been attributed to shell pigmentation or shell patterns without the basic compositional information of the pigment trait itself [38,40]. Similarly, in order to investigate the function and evolution of coloration in avian eggshells and the biochemical pathways that control pigment production, more basic quantitative research on avian eggshell pigmentation is needed, including lineages thus far under-represented in chemical sampling studies [26,42]. The information gap concerning pigment composition in diverse taxa is addressed here by investigating the potential convergence and biochemical basis of color expression in avian eggshells and molluscan shells. To this end, eggshell pigment composition of eleven extinct and extant paleognath avian species, and four molluscan species’ shells (Fig 1) was examined to identify the presence or absence of protoporphyrin and biliverdin and the potential overlap in pigment composition across representatives of diverse phyla (Chordata and Mollusca).

To assess any potential for analogous color production and expression between molluscan shells and avian eggshells it is important to select informative and representative groups. Paleognaths are the most basal group of birds, and the sister taxon to all other modern birds [43]; paleognaths include the flightless ratites and the flying tinamous that are characterized by a primitive complex bone structure in the roof of the mouth (the paleognathous palate) [44]. Paleognaths are ecologically very diverse, distributed mostly throughout the Southern Hemisphere, including its large oceanic islands [45,46] and they comprise less than 1% of extant avian species. Due to their primitive anatomy and exceptional geographical distribution, paleognaths represent a focal group that may assist in unraveling the early evolutionary history of birds [46] and the evolutionary origins of colorful bird eggshells [17,26].

To parallel past and current efforts of pigment characterization in diverse avian lineages, the molluscan samples chosen for this study included representative marine bivalves and gastropods that provide a diverse sampling of colorful mollusks. Bivalves and gastropods are the most common and abundant in terms of species numbers among molluscan groups [47]. Bivalves in particular are one of the most basal groups of mollusks, characterized by a two-halved shell, a burrowing filter-feeding behavior and a wide distribution throughout the world [48]. Gastropods in turn, are characterized by a single (often coiled) shell and also distributed worldwide in virtually all marine and terrestrial habitats [49]. The gastropod species included in this analysis belong specifically to the Neogastropoda, a highly diversified group of active predatory marine snails [50] abundantly found in tropical seas [51]. The molluscan species included here exhibit a wide diversity in shell coloration (Fig 1m–1p) and represent diverse evolutionary histories (bivalves and gastropods) and different ecological strategies (filter-feeding and predation). Although evolutionarily distinct, avian and molluscan shells are composed of a comparable calcium carbonate matrix [13–15], and have similar colors that suggest similar pigments might be found in both avian eggshells and molluscan shells.

## Material and Methods

### Sample Description

**Fig 2:**
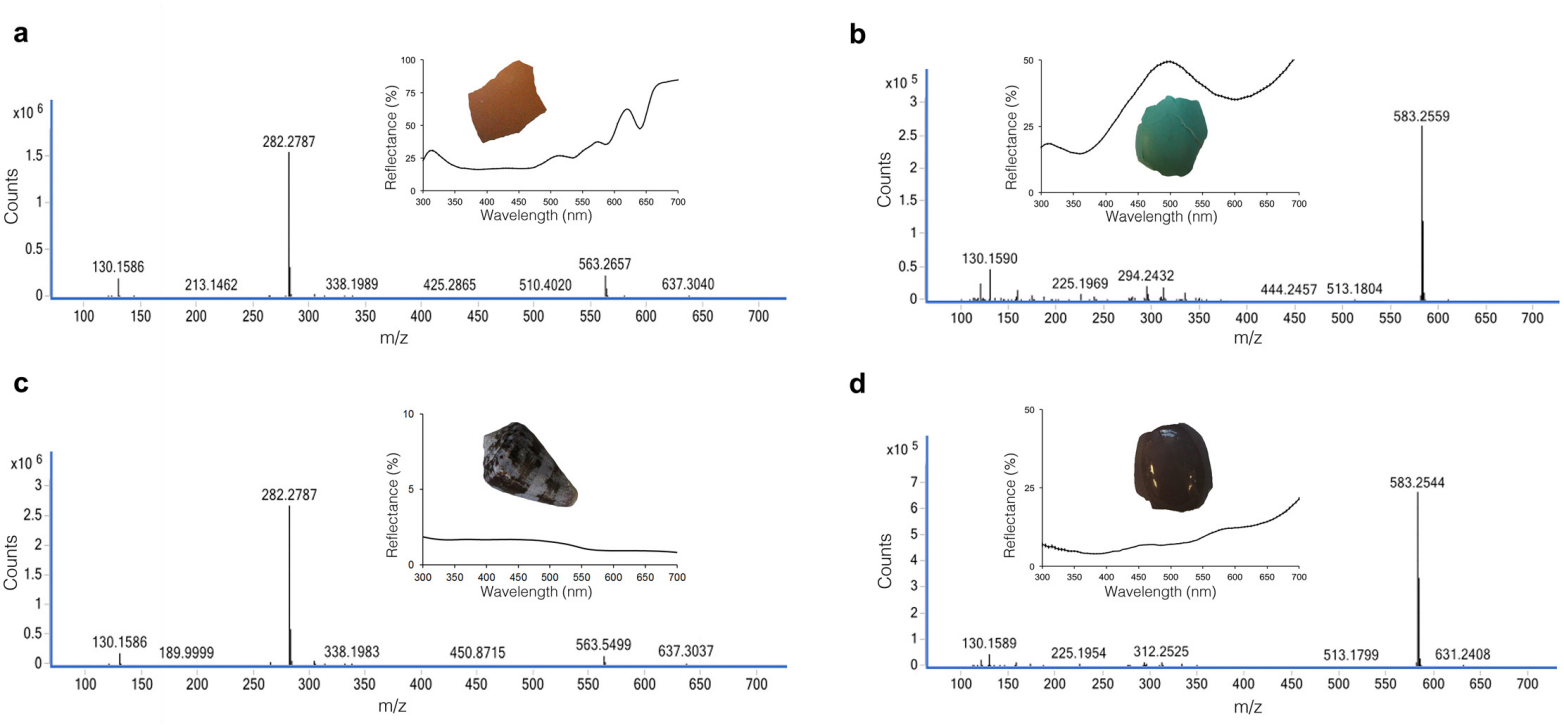
Mass spectrum of pigments extracted from fragments of avian eggshells and molluscan shells. **(a)** Eggshell fragment of positive control *Gallus gallus*, showing protoporphyrin peak (563 m/z) **(b)** eggshell fragment of positive control *Turdus migratorius*, showing biliverdin peak (583 m/z) **(c)** shell fragment of marine snail *Conus purpurascens*, showing protoporphyrin peak (563 m/z) **(d)** shell fragment of *Nothura maculosa*, showing biliverdin peak (583 m/z). Insets: respective reflectance spectra and shell images.

Pigments were extracted from eleven avian eggshells of extinct and extant species of paleognaths. The extinct bird samples included a beige subfossil eggshell sample attributed to a moa (*Euryapteryx curtus*) from Tokerau Beach, New Zealand (Auckland Museum, LB12048) [17] and a beige eggshell sample attributed to an elephant bird (*Aepyornis* sp.), purchased from a commercial source (Bone Room, Solano Street, Berkeley, CA, USA). Both specimens were destructively sampled (i.e. destroyed) as part of the research procedure and are not longer available in a permanent repository. However, equivalent fragments from eggshells of the same species are available upon request. Eggshells of extant paleognaths analyzed comprised five ratites, including ostrich (*Struthio camelus*), greater rhea (*Rhea americana*), emu (*Dromaius novaehollandiae*), Southern cassowary (*Casuarius casuarius*), and North Island brown kiwi (*Apteryx mantelli*); and four species of tinamous, including Chilean tinamou (*Nothoprocta perdicaria*), elegant crested tinamou (*Eudromia elegans*), great tinamou (*Tinamus major*), and spotted nothura (*Nothura maculosa*) (Fig 1a–1k). All extant samples were from captive breeding facilities, including zoos and conservation management operations. These paleognath birds display a wide diversity of human-perceived eggshell color, including white, green, brown and blue, and also have avian-perceivable ultraviolet (UV) peaks (Fig 2). Five additional vertebrate species were included in the analysis as positive and negative controls for known pigmentation. The white immaculate eggshell of the American alligator (*Alligator mississippiensis*) served as a negative control representing a non-avian reptile with a pigment-free eggshell (Fig 1l), while positive avian controls were the neognaths American robin (*Turdus migratorius*), as a known source of biliverdin and the Japanese quail (*Coturnix japonica*), brown-headed cowbird (*Molothrus ater*), and brown egg of the domesticated chicken (*Gallus gallus*) as known sources of protoporphyrin [52]. All collection protocols were approved by the Hunter College Institutional Animal Care and USE Committee #MH 2/16-02. Avian eggshell and molluscan shell purchase or collection localities are specified in S1 Table.

Pigments were also extracted from diversely colored shells from four species of marine mollusks, including the gastropods *Conus ebraeus* from Okinawa (Japan); *Conus purpurascens*, and *Hastula hectica* from Las Perlas Archipelago (Panama); and the bivalve *Argopecten* sp. also from Las Perlas Archipelago (Panama) (Fig 1m–1p). Each species was represented by a single shell fragment. The data were generated to assess the presence or absence of protoporphyrin and biliverdin. While other pigments may have been present, they were not targeted for this study.

Avian eggshells and molluscan shells were broken into smaller fragments (surface area > 1cm and/or weight > 400mg) and subsequently rinsed with distilled water, 70% ethanol to remove any external dirt, and allowed to air dry. Each fragment was then photographed in JPEG format (Nikon Coolpix 8700) under controlled light conditions. We placed a size standard within each photograph and used ImageJ 1.40 (National Institute of Health, USA) to size-calibrate each image and measure the surface area. In addition, the mass of each fragment was measured with a 1 mg precision (Mettler Toledo XS403S) and the thickness measured to an accuracy of 0.01 mm with a point micrometer [53].

Avian eggshell and molluscan shell reflectance was measured between 300–700 nm to document physical appearance [16], using an Ocean Optics Jaz spectrometer and illuminated by a pulsed xenon light source (Ocean Optics, Inc. Dunedin, FL, USA) relative to a commercial white standard (Spectralon, WS-1, Ocean Optics Inc., Dunedin, FL, USA). Six spectra were recorded from various positions on each sample fragment at a 45° coincident oblique measurement geometry to avoid specular glare from the glossy surfaces.

### Pigment Extraction and Quantification

For both avian eggshells and molluscan shell samples, an ethylenediaminetetraacetic acid (EDTA) pigment extraction protocol was followed, as it has been applied in previous pigment studies of avian eggshells (e.g. [19]). This method was milder than the sulphuric acid based protocol used previously [17] to quantify pigments from extinct and extant ratites (following Kennedy and Vevers 1976). We initially tested the acid-based method in the mollusk samples, but due to their thicker shells and consequent higher calcium carbonate content, it proved ineffective to successfully extract pigments for characterization. For this reason, and to compare pigment concentrations obtained by the same extraction method for both groups, we applied the EDTA based protocol [19]. Samples were homogenized by grinding and mashing, and placed in 1.5 ml Eppendorf tubes. Subsequently, 1 ml of aqueous disodium EDTA pH 7.2 (100mg/ml) was added, tubes vortex-mixed for 1 min and uncapped to release pressure. Tubes where then centrifuged at 15,000g for 30 s (Eppendorf 5430R Centrifuge) and the supernatants discarded. This procedure was repeated three times. Sample fragments in the EDTA solution were allowed a contact time of 5 min and briefly vortex-mixed after each repetition. Next, 1 ml of acetonitrile-acetic acid (4:1 v/v) was added, the tubes vortex-mixed for 2 min in 30 s bursts (releasing the stoppers to allow escape of CO2), and subsequently centrifuged for 2 min at 15,000g. The supernatants were then transferred to clean tubes and stored at 4°C in the dark until further analysis within 24h.

An aliquot was measured in a NanoDrop 2000c spectrophotometer for its UV-Vis absorbance spectrum from 250–700 nm versus acetonitrile-acetic acid as a blank. Pigment presence was indicated from these spectra and confirmed and quantified by High Performance Liquid Chromatography (UHPLC) and Mass Spectrophotometry (MS) (S1 Fig). UHPLC analysis was performed as described in [19]. To summarize, samples were run with a flow rate of 1.00 mL/min using water with 0.05% Trifluoroacetic acid (TFA) and acetonitrile as solvents A and B, respectively. The elution gradient used was 90% A, 10% B with a linear gradient to 0% A and 100% B at 25min. Solvent B was then run isocratically for a further 10 min. Absorbance was monitored at 377 nm for the first 15 minutes, then switched to 405 nm for the remainder of the gradient. Biliverdin eluted at ~3.7 min and protoporphyrin at ~6.1 min. Samples in which a pigment was not identified with this method, were analyzed by mass spectrometry to detect the presence of biliverdin and protoporphyrin through a “by formula” search as described below.

When detected, pigment concentration was standardized by the mass (g^−1^) of the sample fragment [17,18]. For comparison, and taking into account the fragment thickness, we additionally standardized pigment concentration by volume (mm^−3^) (Table 1). These are reliable measurements of pigment concentration if pigment is deposited throughout the entire depth of the eggshell or molluscan shell matrix [15,18]. However, it has been reported for some avian species that the majority of pigmentation occurs within the eggshell cuticle [54] and consequently, we also standardized pigment concentration by surface area of the sample fragment (cm^−2^) [17]. In addition, all observed pigments were compared to commercially obtained standards of biliverdin and protoporphyrin, and the respective extinction coefficient of biliverdin (56200 M^-1^cm^-1^) and protoporphyrin (171000 M^-1^cm^-1^) was used to calculate concentrations in our test samples (Frontier Scientific, Inc. UT, USA). Solutions of commercially obtained biliverdin and protoporphyrin were analyzed by UV-Vis absorbance spectra, UHPLC and Mass Spectrometry using the same methods applied to the avian and molluscan samples.

**Table 1:** Relative concentration of biliverdin and protoporphyrin in avian eggshells in which pigment was detected.

Species^a^	Pigment	Absorbance^b^	Concentration		
			(nmol g^-1^)	(nmol mm^-2^)	(nmol mm^-3^)
*Dromaius novaehollandiae*	Biliverdin	0.6	18.6	46.7	53.6
*Casuarius casuarius*	Biliverdin	1.0	25.5	64.6	67.3
*Nothoprocta perdicaria*	Biliverdin	1.0	41.5	23.5	134.4
*Eudromia elegans*	Biliverdin	0.5	16.0	10.3	62.3
*Tinamus major*	Biliverdin	0.2	4.5	3.3	12.8
*Nothura maculosa*	Biliverdin	0.5	15.3	10.1	50.4
*Turdus migratorius*	Biliverdin	0.4	97.1	28.1	351.9
*Molothrus ater*	Biliverdin	0.7	120.9	46.1	1537.4
*Coturnix japonica*	Biliverdin	1.4	62.3	25.6	150.4
*Molothrus ater*	Protoporphyrin	1.2	57.9	22.1	736.4
*Coturnix japonica*	Protoporphyrin	1.5	21.0	8.6	50.6
*Gallus gallus*	Protoporphyrin	1.1	9.3	8.5	25.8

^a^Paleognaths appear on the first half of the table and neognaths on the second half.

^b^Biliverdin absorbance measured at 377 nm; protoporphyrin absorbance measured at 405 nm.

## Results

An EDTA pigment extraction technique allowed the successful detection and characterization of biliverdin from eggshells of *D*. *novaehollandiae*, *C*. *casuarius* and, for the first time, from colorful eggshells of the four tinamou species *N*. *perdicaria*, *E*. *elegans*, *T*. *major* and *N*. *maculosa*. Protoporphyrin was the only pigment detected from molluscan shells of *H*. *hectica*, *C*. *ebraeus*, *C*. *purpurascens* and *Argopecten* sp. (Table 2). Either biliverdin or protoporphyrin, or both pigments, were detected in all of the positive control species. Biliverdin was detected from the eggshells of *T*. *migratorius*, *M*. *ater* and *C*. *japonica*, and protoporphyrin was detected from eggshells of *G*. *gallus*, *M*. *ater* and *C*. *japonica*. The negative control, *A*. *mississippiensis*, did not yield any pigments (Table 2). Mass spectrometry analysis of the negative control *A*. *mississippiensis* and bird eggs with only biliverdin detected by UV-Vis absorption spectra did not yield protoporphyrin by UHPLC/MS analyses, confirming that protoporphyrin detected in molluscan shells was not a result of contamination.

**Table 2:** Biliverdin and protoporphyrin presence/absence in shells of avian and molluscan species included in the analysis. Avian species (and a non-avian reptile) appear on the first half of the table and molluscan species appear on the second half.

Species	Pigment	
	Biliverdin	Protoporphyrin
*Euryapteryx curtus* ^†^	-	-
*Aepyornis* sp.^†^	-	-
*Dromaius novaehollandiae*	+	-
*Casuarius casuarius*	+	-
*Rhea americana*	-	-
*Struthio camelus*	-	-
*Apteryx mantelli*	-	-
*Nothoprocta perdicaria*	+	-
*Eudromia elegans*	+	-
*Tinamus major*	+	-
*Nothura maculosa*	+	-
*Turdus migratorius*	+	-
*Molothrus ater*	+	+
*Coturnix japonica*	+	+
*Gallus gallus*	-	+
*Alligator mississippiensis*	-	-
*Hastula hectica*	-	+
*Conus purpurascens*	-	+
*Conus ebraeus*	-	+
*Argopecten* sp.	-	+

Pigment in the EDTA extract was first evaluated by UV-Vis absorption spectra. Biliverdin was indicated by an absorbance peak at 376 nm in eggshell extracts of *D*. *novaehollandiae*, *C*. *casuarius*, *N*. *perdicaria*, *E*. *elegans*, *T*. *major* and *N*. *maculosa* plus *T*. *migratorius* (positive control). Protoporphyrin was indicated by an absorbance peak at 400 nm only in eggshell extracts of the positive controls, *G*. *gallus*, *M*. *ater* and *C*. *japonica*.

Biliverdin and protoporphyrin pigments were confirmed and quantified by UHPLC and MS. Chromatograms of representative species including the tinamou *N*. *maculosa* and the gastropod *C*. *purpurascens* are shown in Fig 2. The *N*. *maculosa* eggshell extract shows a peak at 583 m/z, consistent with a free acid biliverdin molecule. The *Conus purpurascens* shell extract chromatogram shows a peak at 563 m/z that corresponds to a free acid protoporphyrin molecule. However, the concentration was below the detection threshold for UV-Vis spectroscopy, and the presence of protoporphyrin was confirmed through MS ion detection at specific masses as described below. All UHPLC/MS chromatograms are included in S1 Fig.

UV-Vis spectroscopy failed to detect protoporphyrin, biliverdin IX or both, in the shells of several species, including some paleognaths and all mollusks. As a result, the extracts of these species were analyzed through MS ion detection at specific masses, present at 563 m/z and 583 m/z respectively. With this method, protoporphyrin was detected in all molluscan shells. Pigment concentrations detected for all species are shown in Table 1. Protoporphyrin concentration of molluscan shells lies below the UHPLC detection threshold and cannot be estimated.

We further analyzed all samples, including the negative control (i.e. alligator egg), through MS ion detection to ensure detection of all pigments that might be below the detection threshold of UV-Vis spectroscopy or UHPLC. Thus, the protoporphyrin samples were searched for the input mass of 583 m/z (biliverdin) and biliverdin samples were analyzed at an input mass of 563 m/z (protoporphyrin). In both cases, negative results were obtained, similar to the alligator egg negative control.

## Discussion

In an effort to examine pigment composition across phyla, colorful shells of eleven species of paleognath birds, including all but one of known extinct and extant lineages, and four species of mollusks were analyzed (Fig 1). Both avian eggshells and molluscan shells share a similar calcium carbonate structure [13–15], but it was previously unclear if these structures also possess similar pigments. Our findings suggest that there is a shared chemical similarity in pigments across avian and molluscan taxa, as tetrapyrrole pigments were detected in both phyla (Chordata and Mollusca). Biliverdin and protoporphyrin were both detected in avian taxa, whereas in mollusks only protoporphyrin was identified. The modified [19] extraction and identification protocol applied allowed the detection and quantification of biliverdin in six species of paleognaths, including *D*. *novaehollandiae*, *C*. *casuarius*, and for the first time, in the tinamou species *N*. *perdicaria*, *E*. *elegans*, *T*. *major* and *N*. *maculosa* (Table 2). In addition, only protoporphyrin was detected from molluscan species, including *H*. *hectica*, *C*. *ebraeus*, *C*. *purpurascens* and *Argopecten* sp. (Table 2). Previous studies of molluscan shells reported mostly uroporphyrin [32,33,37,38]; however, we have identified another porphyrin molecule from mollusk shells: protoporphyrin. Although previously reported at trace levels [17], protoporphyrin was not detected for *Rhea americana*, *E*. *curtus* and *S*. *camelus* eggshells in our study. This discrepancy may have been due to the EDTA extraction method used [19], as this method is less corrosive and does not use strong acids typical of other avian extraction protocols [17]. Based on our findings, we suggest that EDTA extraction alone should not be relied on for determining molluscan shell pigment concentrations. The quantity of protoporphyrin detected in molluscan shells was insufficient to provide absorbance measurements and could only be identified by the more sensitive mass spectrometry analyses. It would appear that snail shell pigment extraction requires more stringent methods. However, our attempts using strong acid [17] resulted in an extract unsuitable for absorbance detection. Further studies are necessary to identify an optimal snail shell extraction method, and therefore our results do not reflect all the pigments that may be present in molluscan shells.

Paleognath eggs were chosen for this study as these birds are basal and the sister group to all modern birds and any investigation into their pigment composition will yield substantive information concerning pigment evolution in birds. Despite the great potential of paleognaths to reveal the evolution of phenotypic traits in birds, only a handful of studies exist investigating the pigmentation [17,19] and coloration [31] of paleognath eggshells, including subfossil shells of now extinct moas, [17, this study] and elephant birds (this study). Among paleognaths, the glossy and colorful eggs of tinamous have been of particular interest [31,55]. Our analysis successfully detected biliverdin from four species of tinamou using chemical extraction techniques for the first time. This finding is significant as it suggests that even the bright and diverse colors of tinamou eggs are determined by the same pigment(s) already detected in paleognaths [17] and neognaths [19].

As with avian eggshells, the pigment composition of molluscan shells is not well understood and reports on molluscan pigments in the literature are rather limited [13,32,37,38]. Despite the dearth of information on molluscan shell pigments, there are several theories on the control of pigment expression and, in some cases, the pressures acting on this trait. Variation in molluscan shell color has been shown to be under genetic and neuronal control, guided by a local activation and lateral inhibition (LALI) model similar to what is found in the cortical region of mammalian brains [56] or generated by a neural-network model of the mantle [57]. Variations in molluscan pigments may also be related to environmental gradients such as climate, insolation, wave exposure and salinity. In some cases pigment has been associated with differences in growth and survival, suggesting the adaptive significance of shell pigmentation [41,58]. Identifying the pigments responsible of avian eggshell and molluscan shell coloration can shed light on the biological functions, evolutionary origins and control mechanisms of pigmentation.

This study confirms the presence of tetrapyrrole pigments in two distantly related shell-building groups, birds and mollusks, strengthening previous hypotheses about the ubiquitous role of tetrapyrroles in shell coloration in biodiverse organisms. The universal presence of tetrapyrroles across bird lineages has been used to propose an ancient origin and high conservation of these pigments throughout avian evolution [17]. In fact, biliverdin, protoporphyrin, and other tetrapyrroles are produced ubiquitously across all phyla, with a shared porphyrin biosynthesis pathway active in all non-photosynthetic eukaryotes, including animals and fungi [18,20]. Accordingly, protoporphyrin was detected in both avian and molluscan shells suggesting that the mechanisms of pigment production are also shared across different phyla (i.e. Chordata and Mollusca). However, the presence of tetrapyrrole pigments in diverse calcareous shells is not suggestive of evolutionary, developmental, or biochemical linkages between the deposition of these pigments and the production of calcareous shells, such as those made by mollusks or birds. The results presented here suggest that the pigment molecules coloring calcareous shells are highly conserved in nature’s palette and used not only throughout avian evolution, but also in distantly related taxa throughout metazoan evolution, including colorful lineages of marine mollusks.

## Supporting Information

S1 Fig UHPLC/MS chromatograms for pigment extracts from all avian eggshells and molluscan shells included in the study. (PDF)

S1 Table Avian eggshell and molluscan shell purchase or collection locations. (PDF)
