# Supplementary material for: Nature’s Palette: Characterization of Shared Pigments in Colorful Avian and Mollusk Shells
Source: PLoS One. 2015 Dec 9;10(12):e0143545. doi: 10.1371/journal.pone.0143545 (PMC4674117; doi:10.1371/journal.pone.0143545)

Verdes A, Cho W, Hossain M, Brennan PLR, Hanley D, Grim T, Hauber ME, Holford M. **Nature's Palette: Characterization of shared pigments in colorful avian and mollusk shells**. PLoS ONE. Corresponding authors: averdes@gradcenter.cuny.edu, mholford@hunter.cuny.edu

S2 Figure. UHPLC/MS chromatograms for pigment extracts from all avian eggshells and molluscan shells included in the study.

Japanese Quail (at 583)

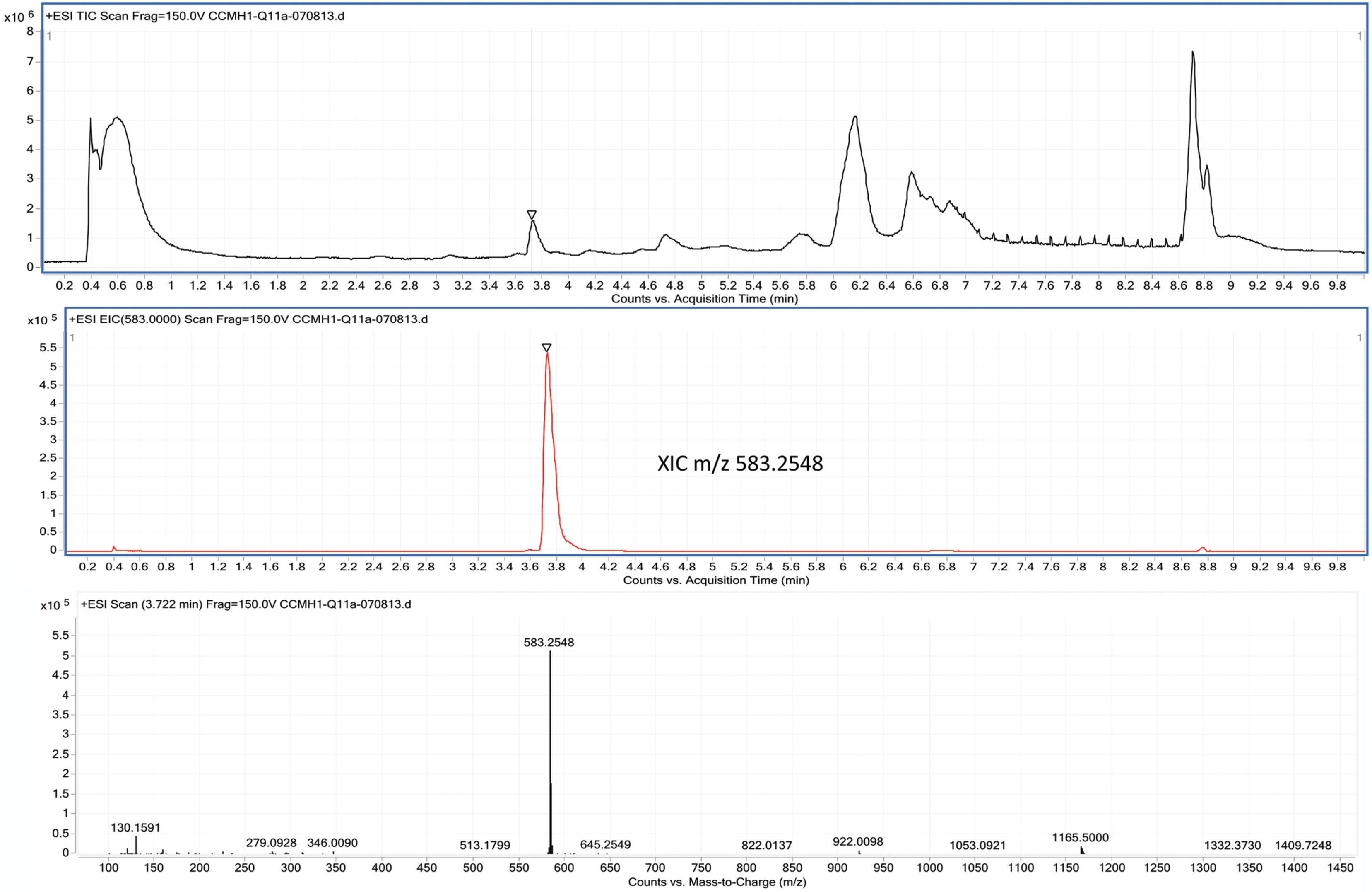

Verdes A, Cho W, Hossain M, Brennan PLR, Hanley D, Grim T, Hauber ME, Holford M. **Nature's Palette: Characterization of shared pigments in colorful avian and mollusk shells**. PLoS ONE.
Corresponding authors: averdes@gradcenter.cuny.edu, mholford@hunter.cuny.edu

ESM_1 UHPLC/MS chromatograms of all taxa included in the analysis

Japanese Quail (at 583)

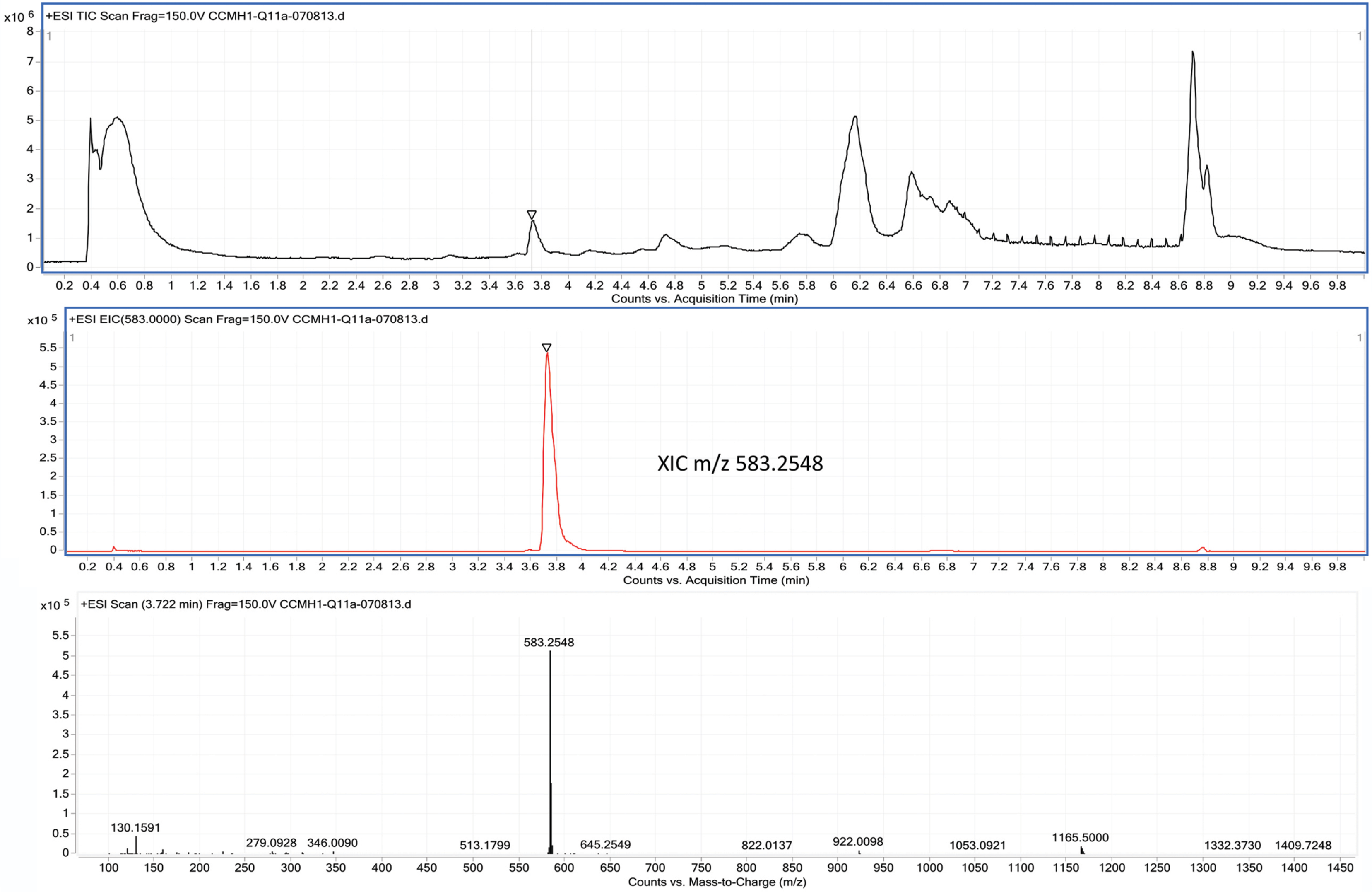

Japanese quail (at 563)

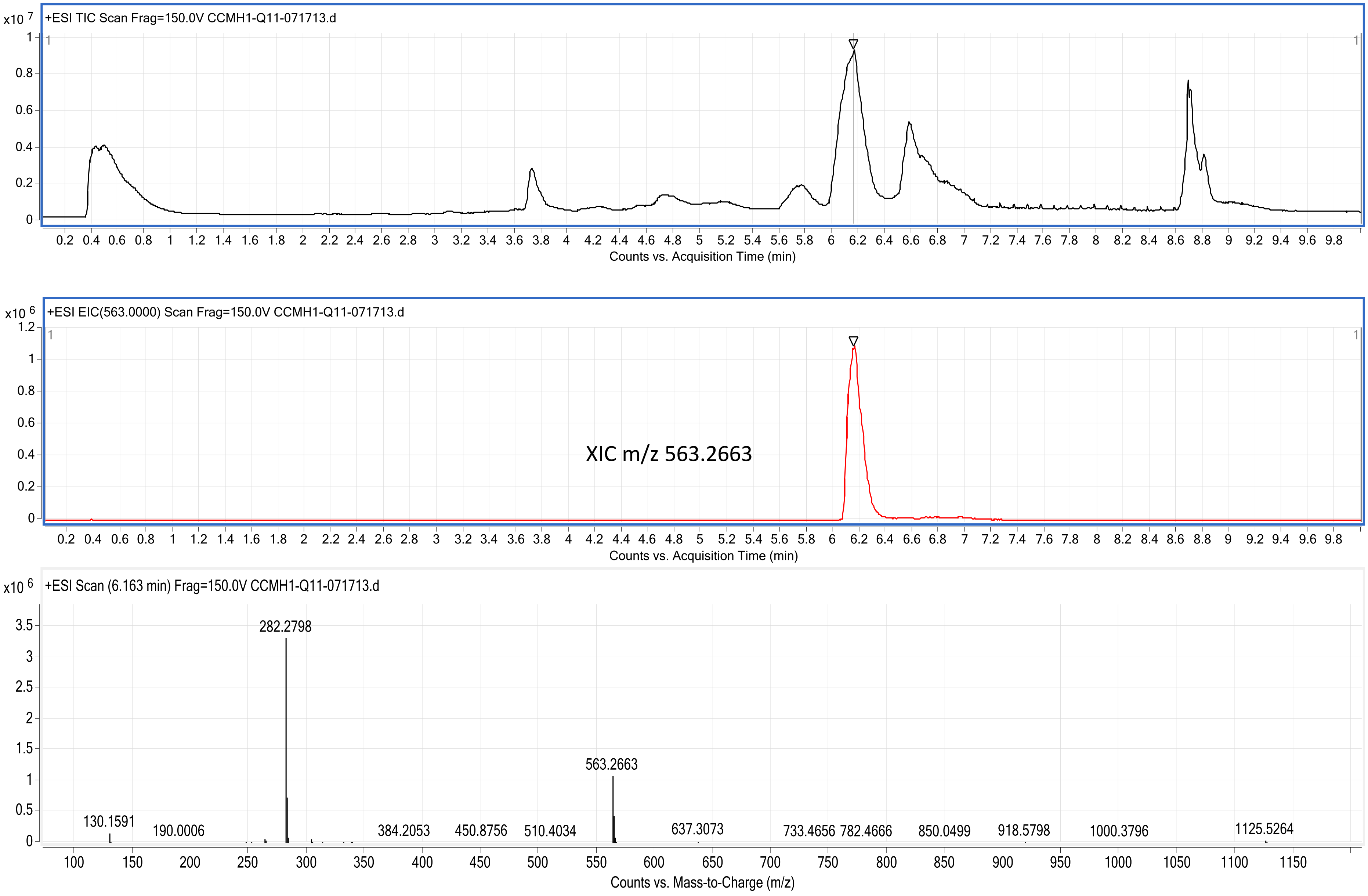

Domesticated chicken (at 563)

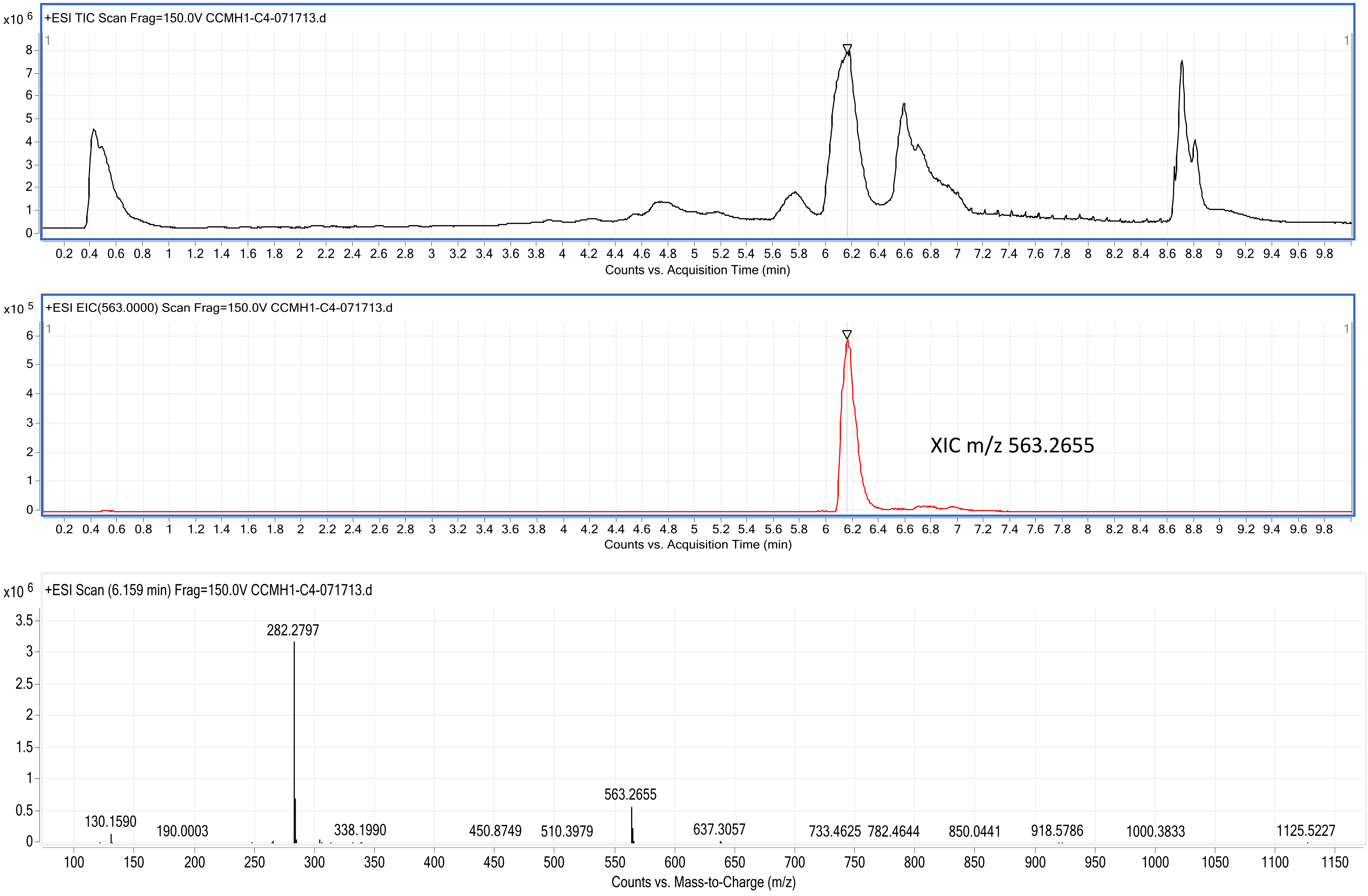

Brown-headed cowbird (at 563)

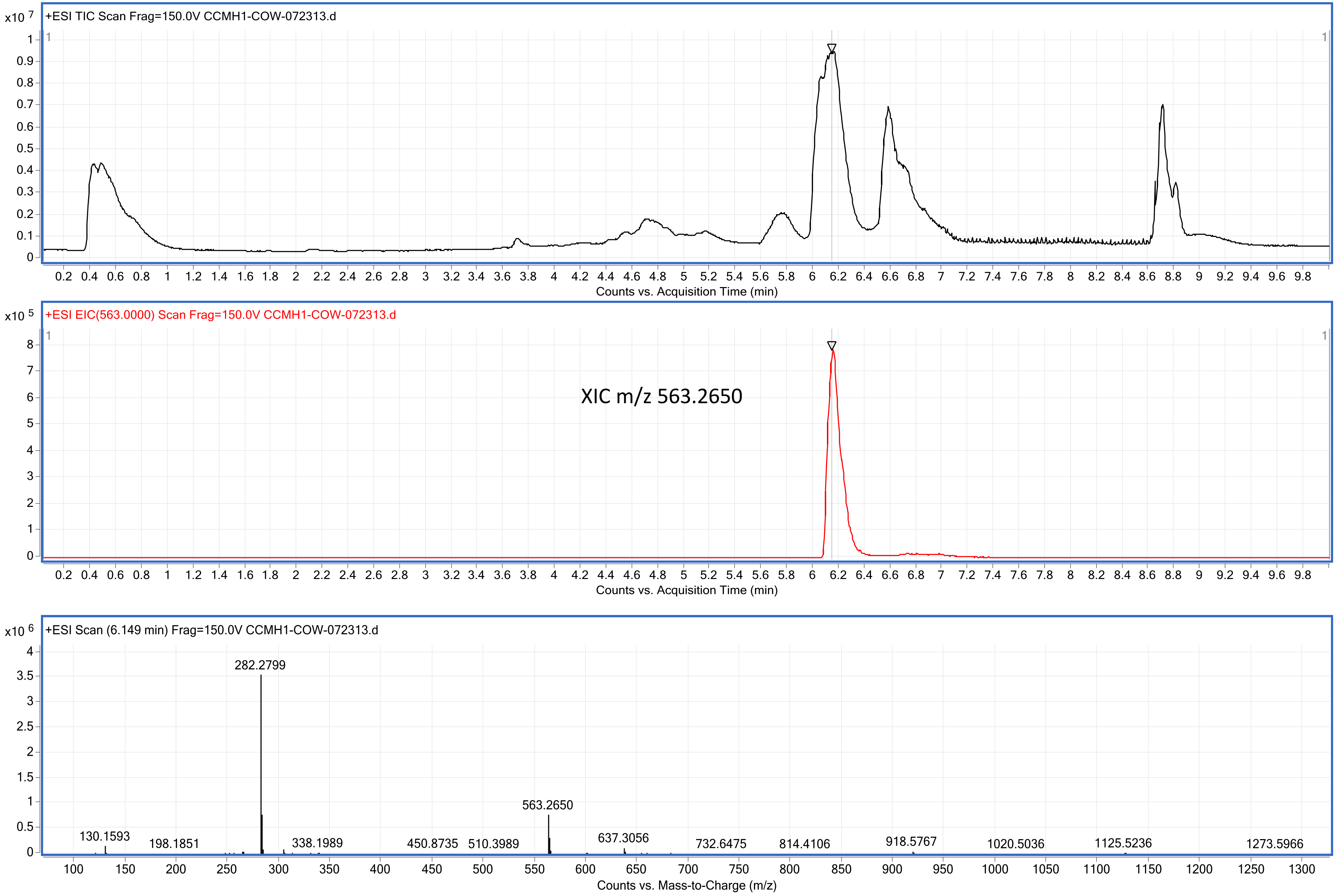

Brown-headed cowbird (at 583)

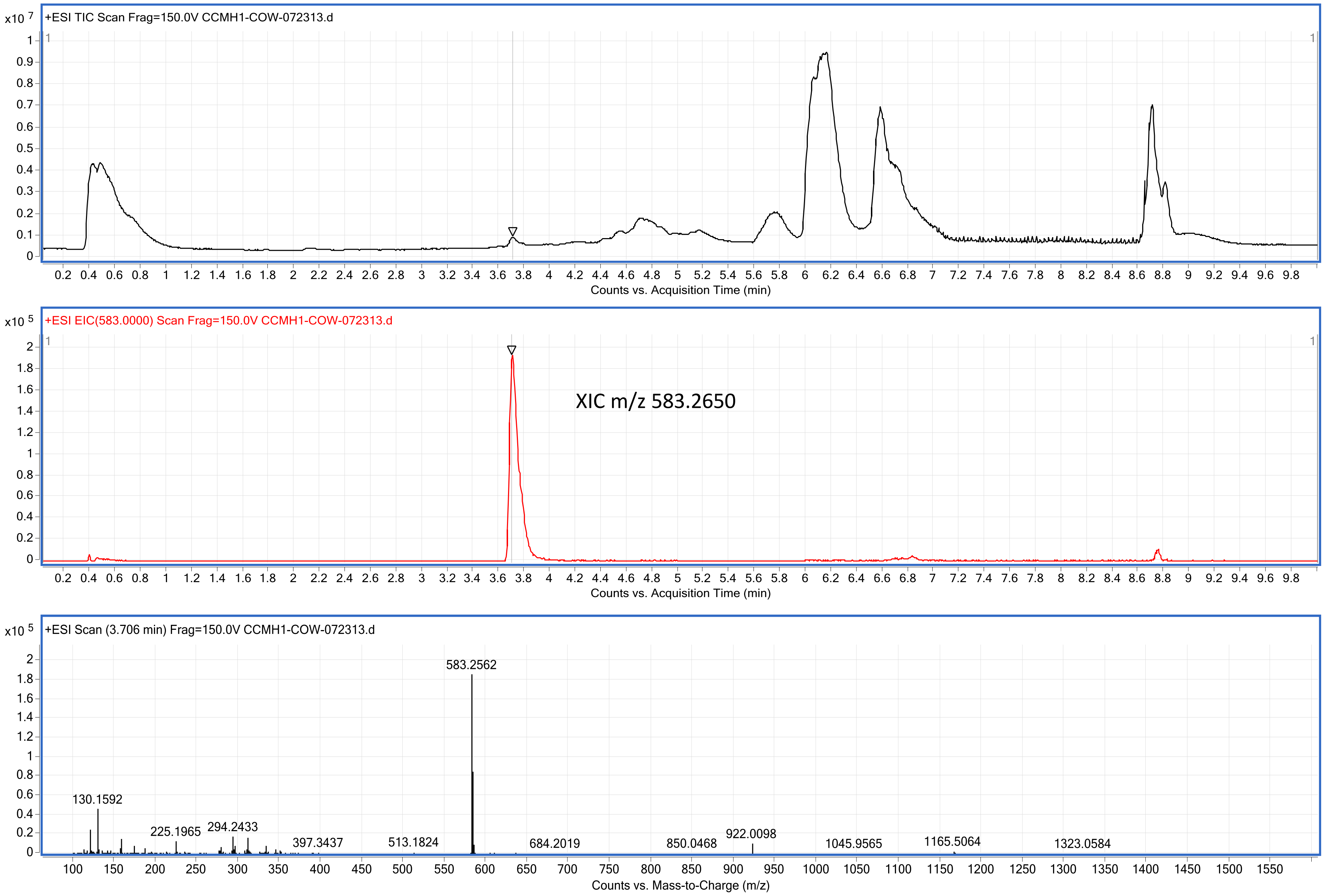

American Robin (at 583)

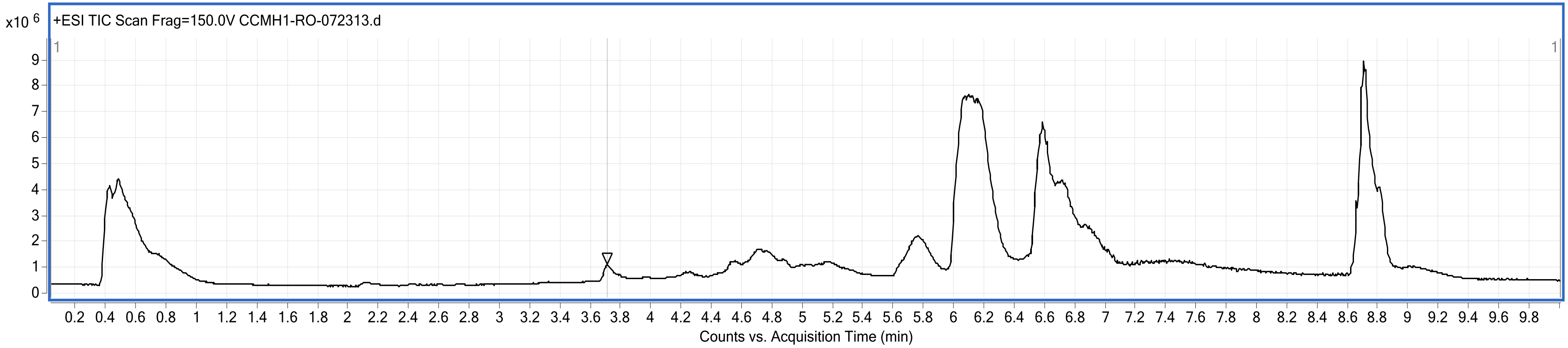

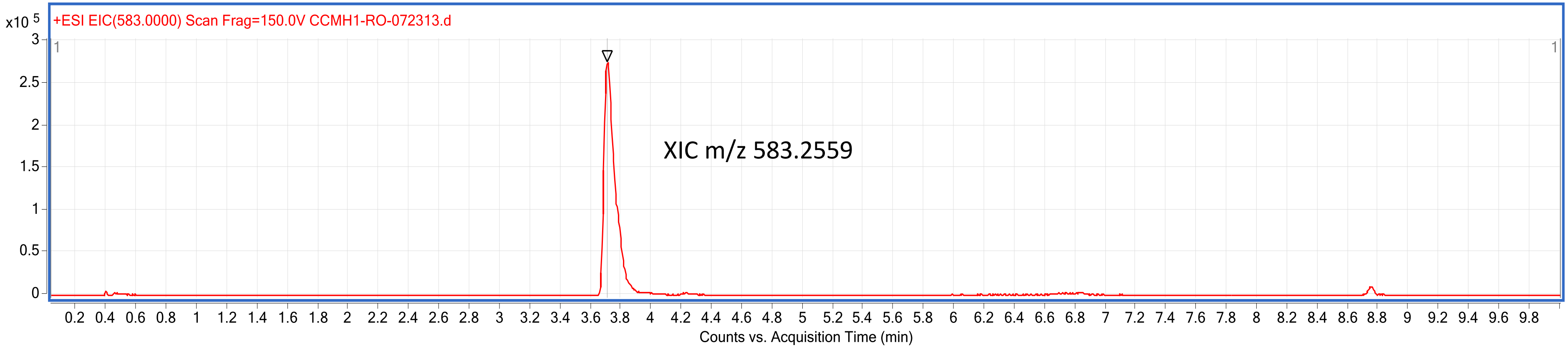

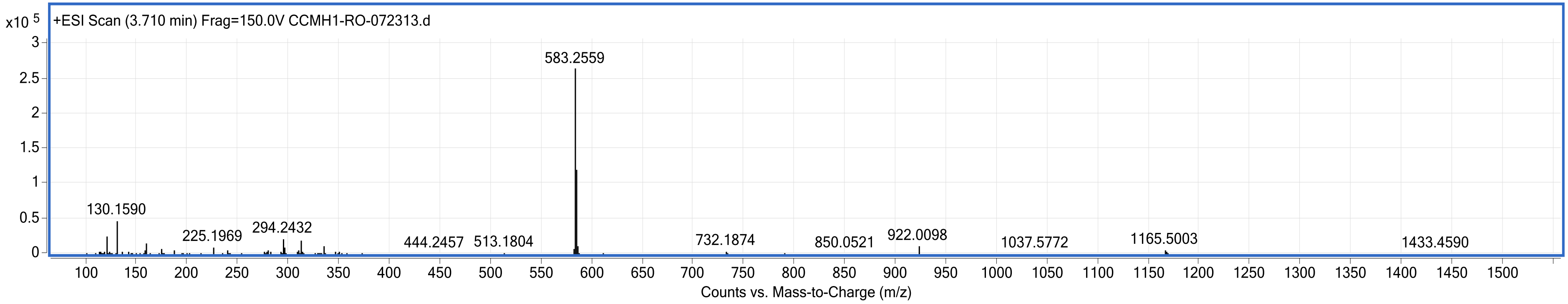

American robin (at 563)

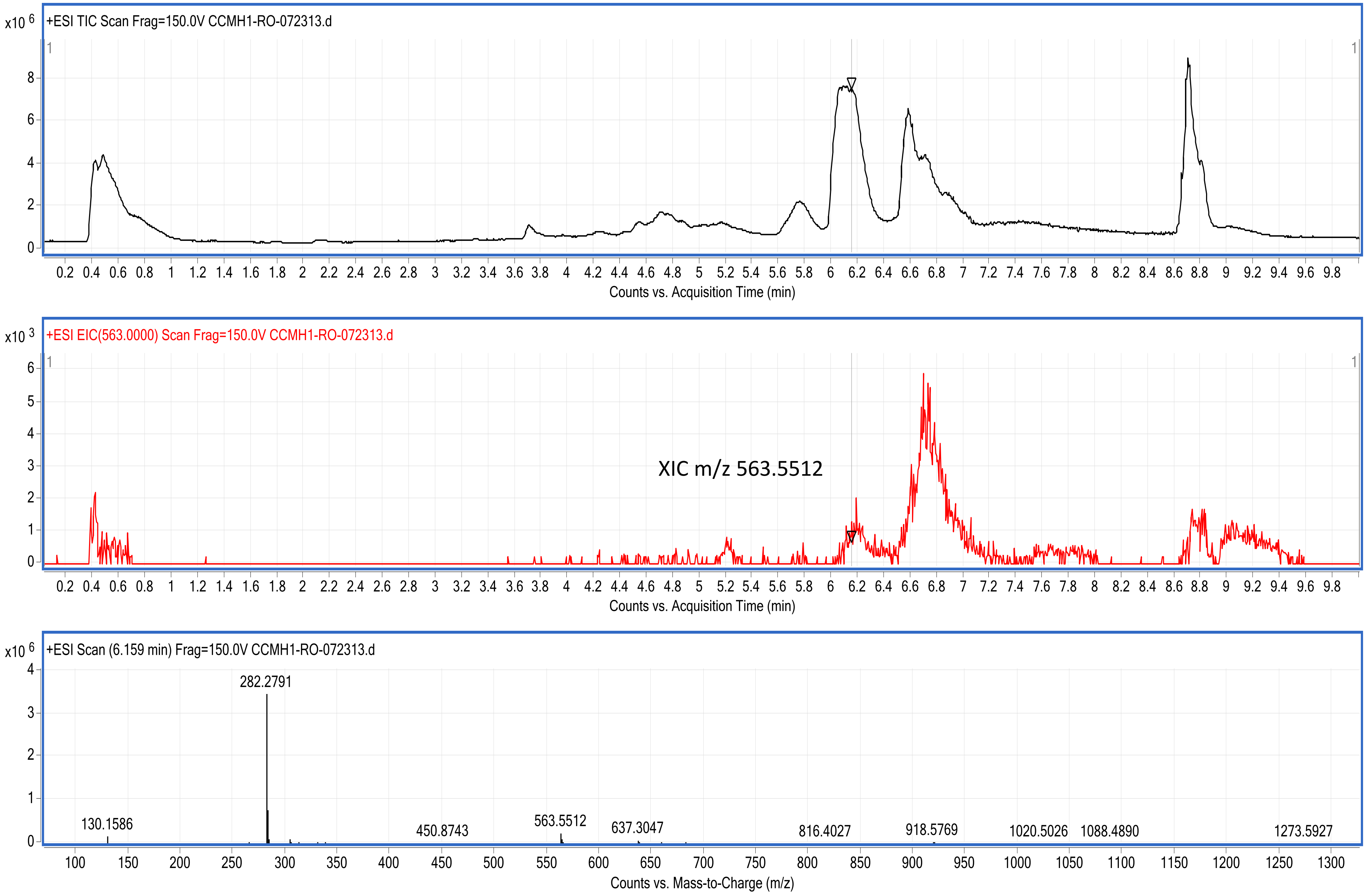

American alligator (at 563)

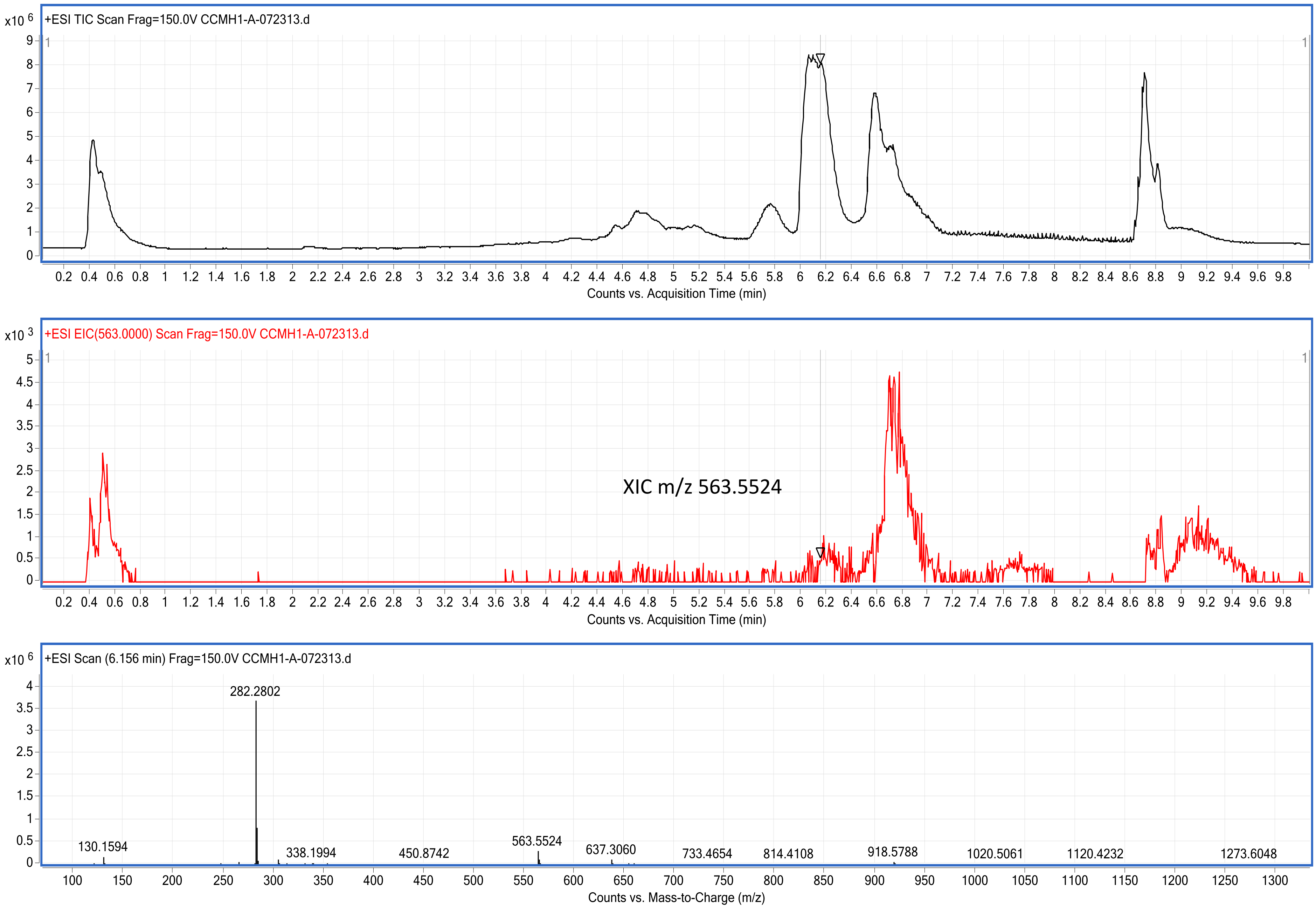

Emu (at 583)

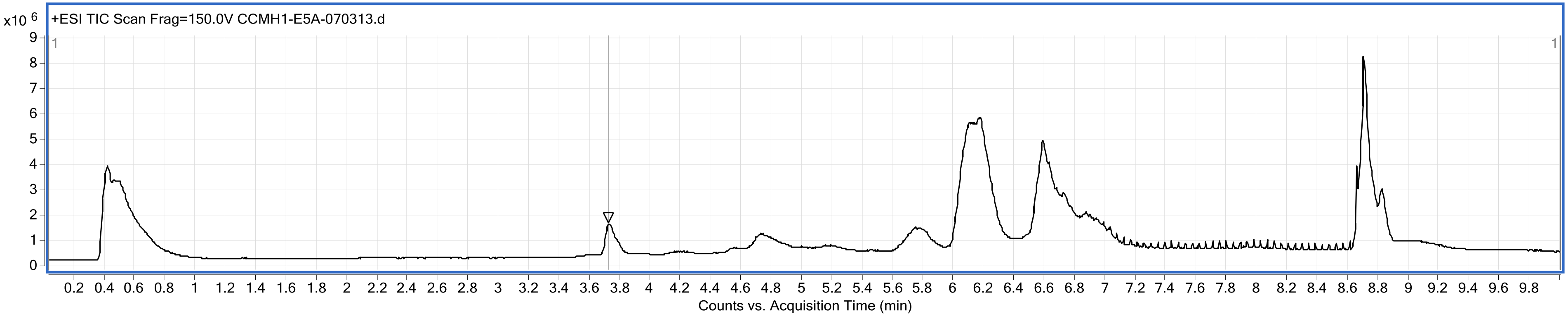

x10 6
+ESI TIC Scan Frag=150.0V CCMH1-E5A-070313.d
Counts vs. Acquisition Time (min)

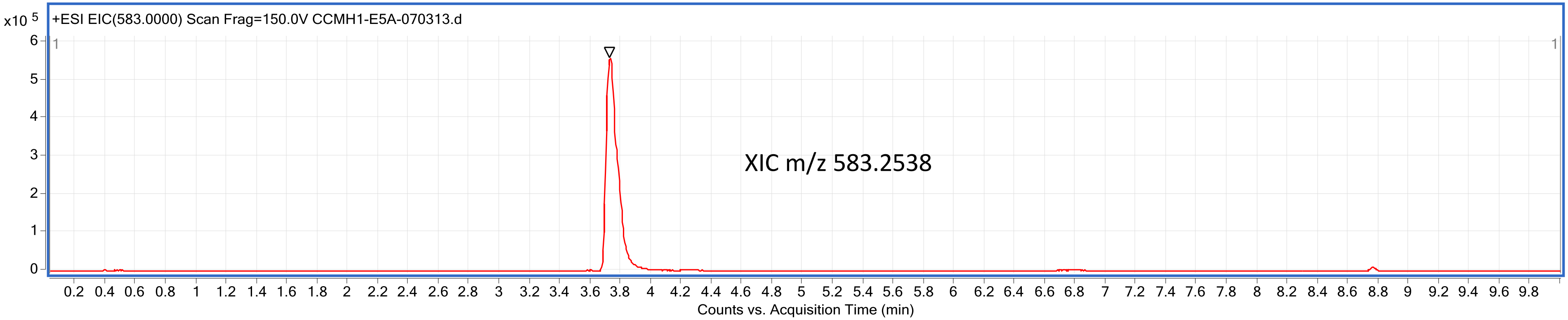

x10 5
+ESI EIC(583.0000) Scan Frag=150.0V CCMH1-E5A-070313.d
XIC m/z 583.2538
Counts vs. Acquisition Time (min)

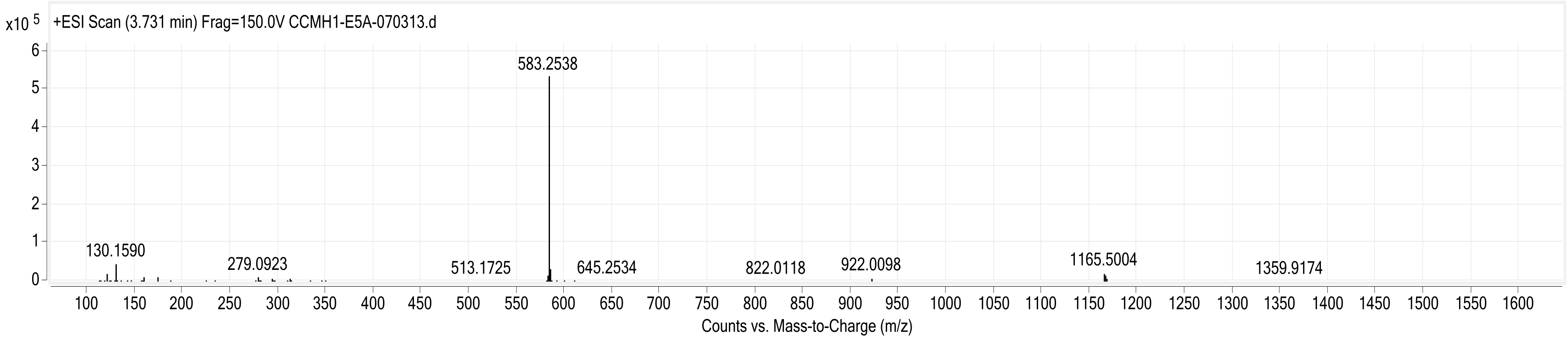

x10 5
+ESI Scan (3.731 min) Frag=150.0V CCMH1-E5A-070313.d
583.2538
130.1590
279.0923
513.1725
645.2534
822.0118
922.0098
1165.5004
1359.9174
Counts vs. Mass-to-Charge (m/z)

Ostrich (at 563)

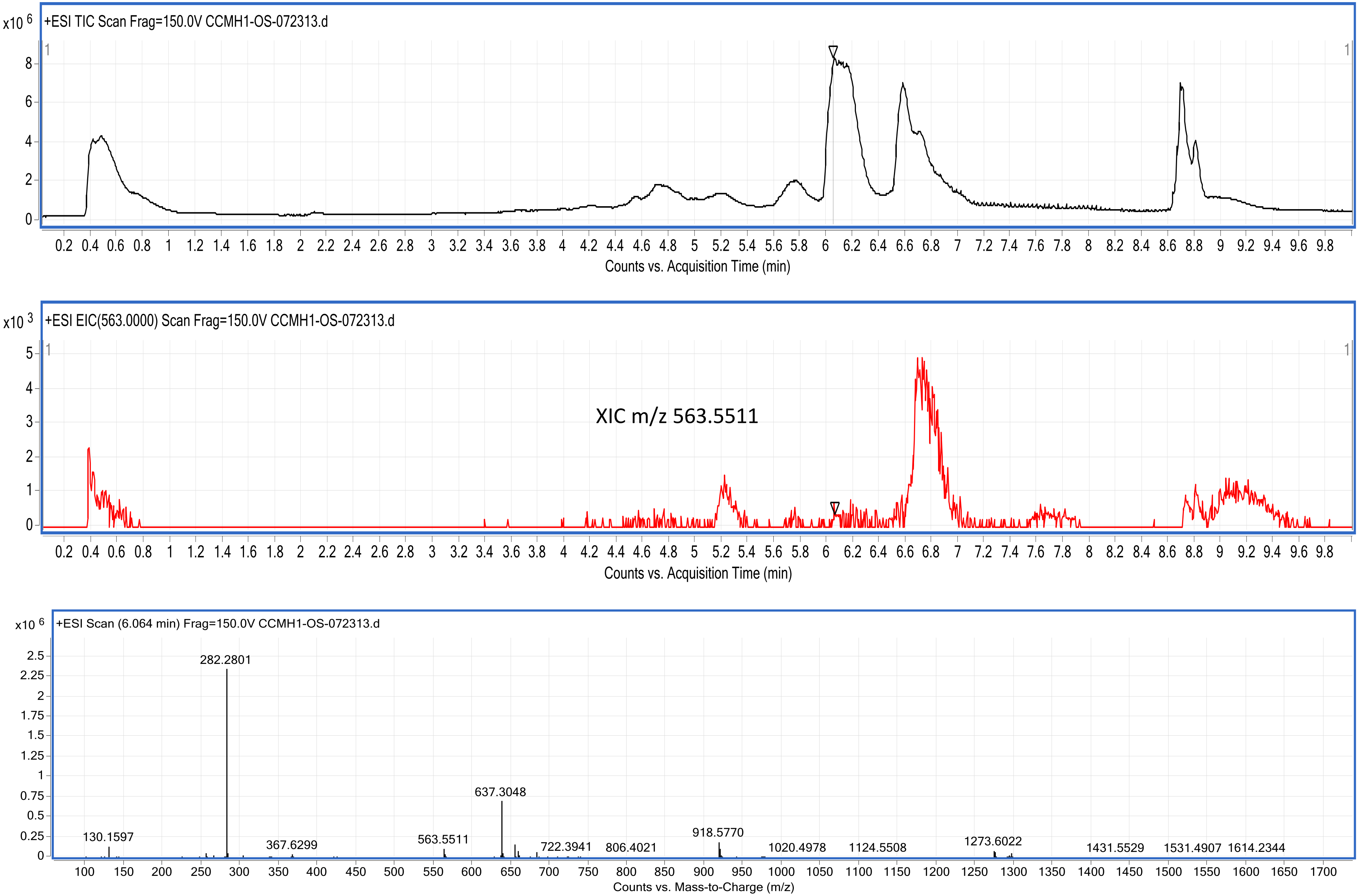

Southern cassowary (at 583)

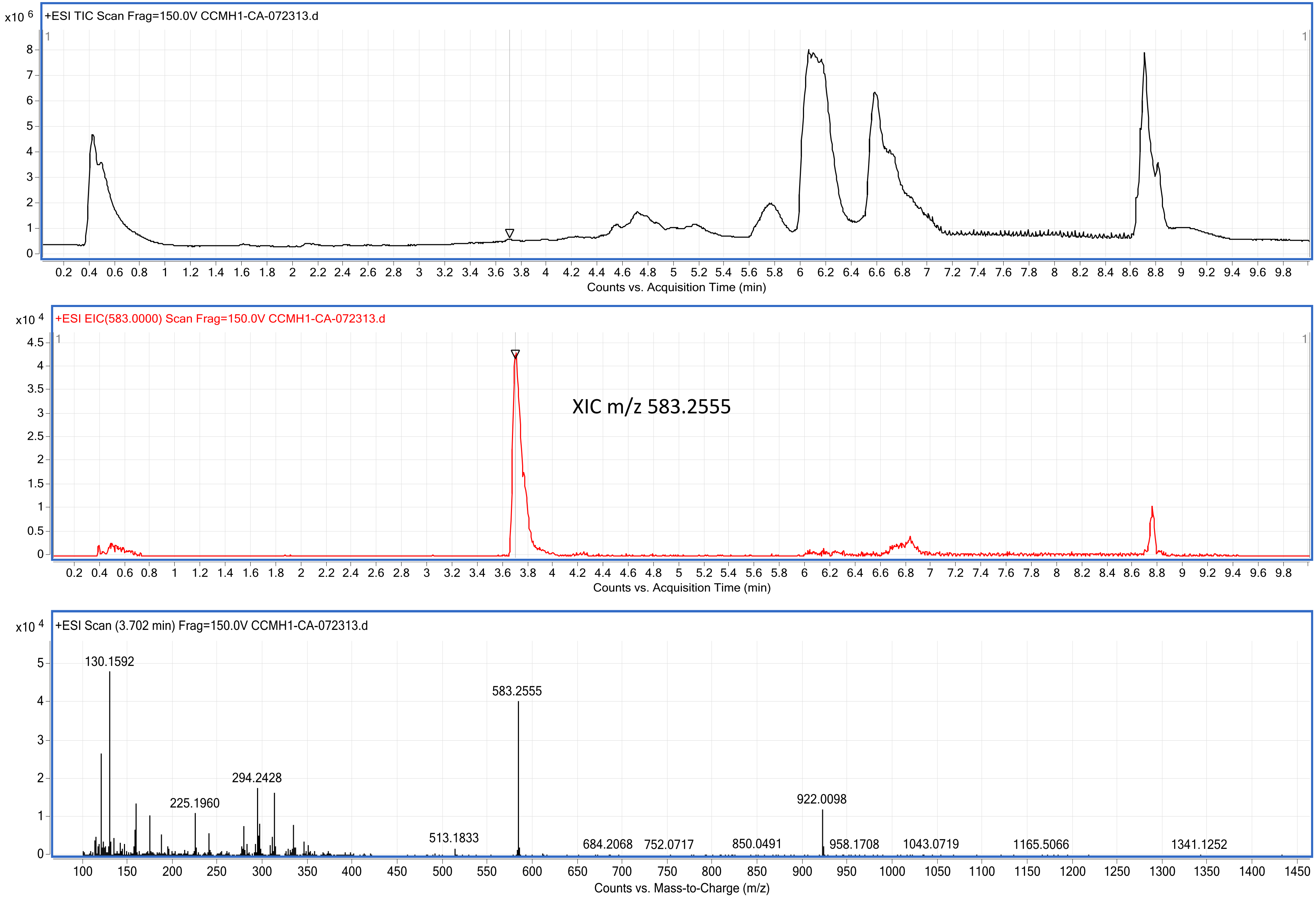

Southern cassowary (at 563)

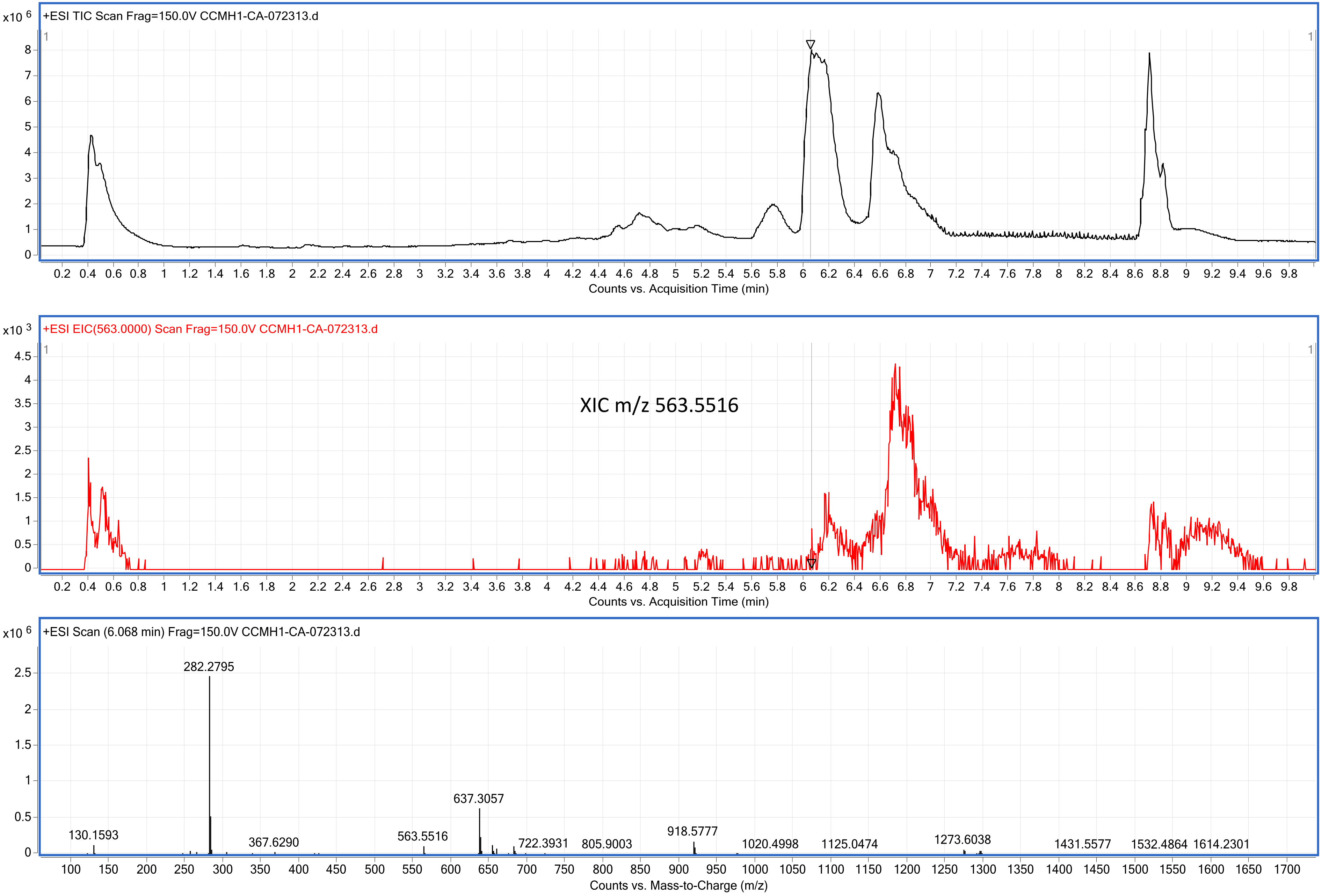

Elephant bird (at 563)

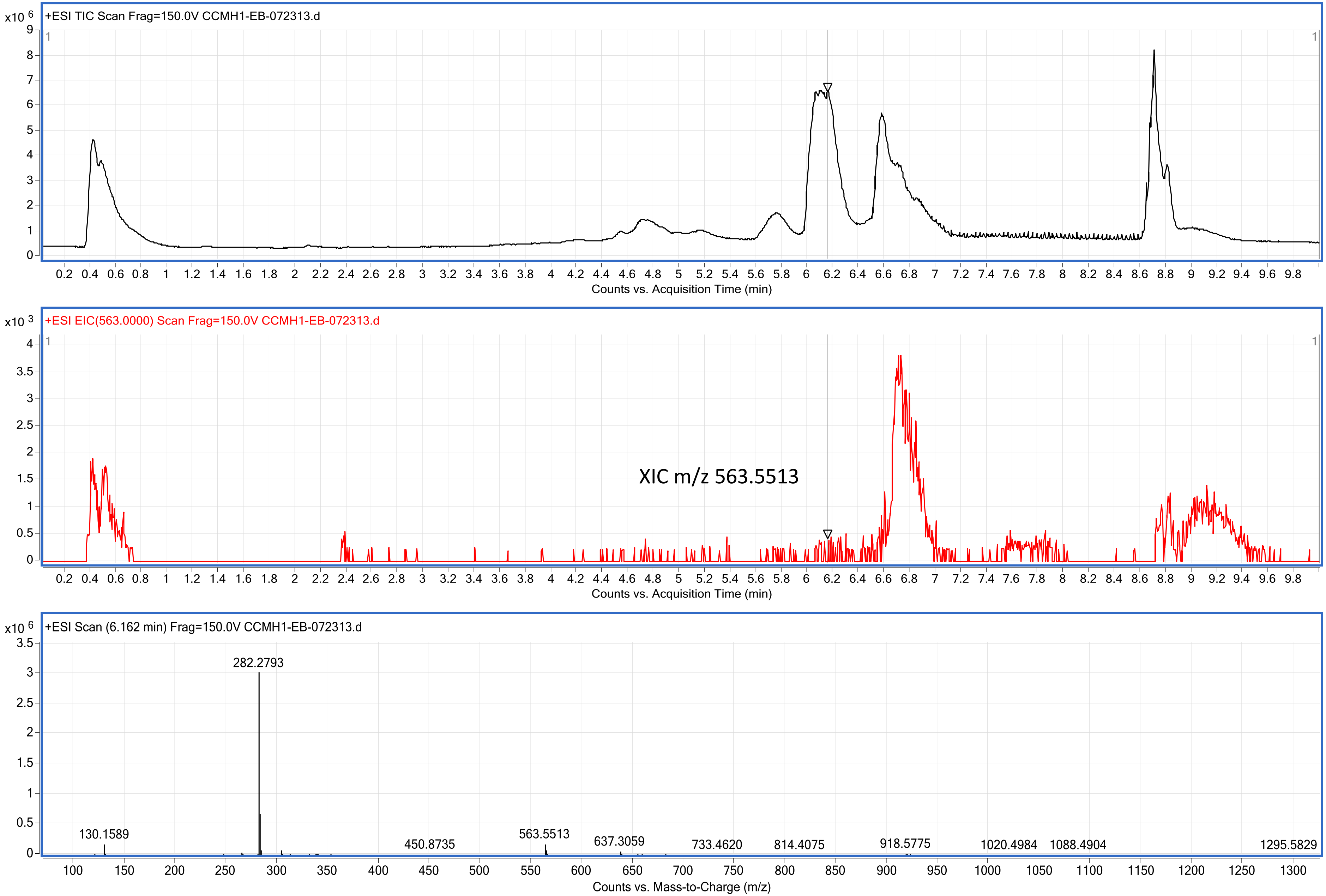

North Island brown kiwi (at 563)

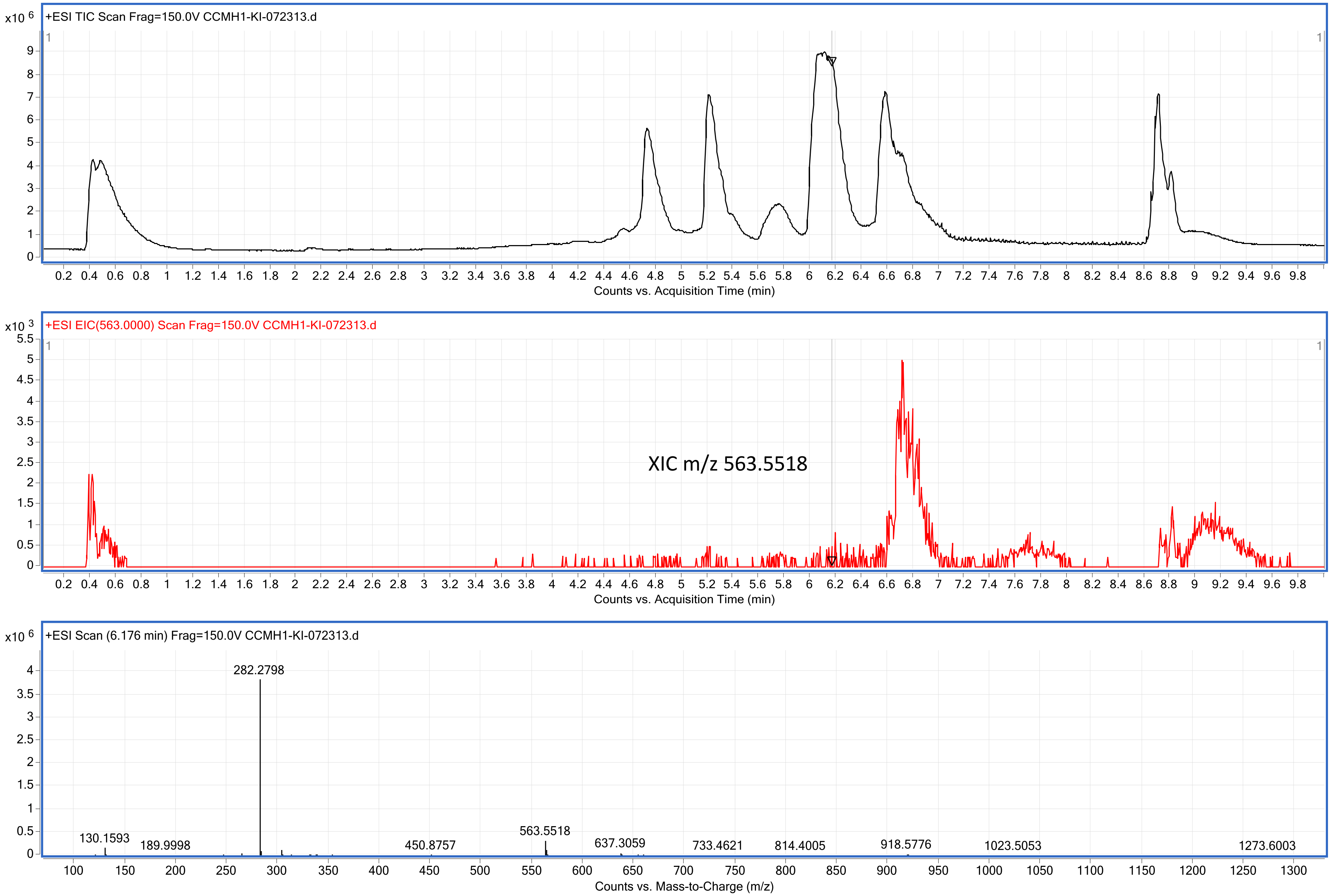

Moa (at 563)

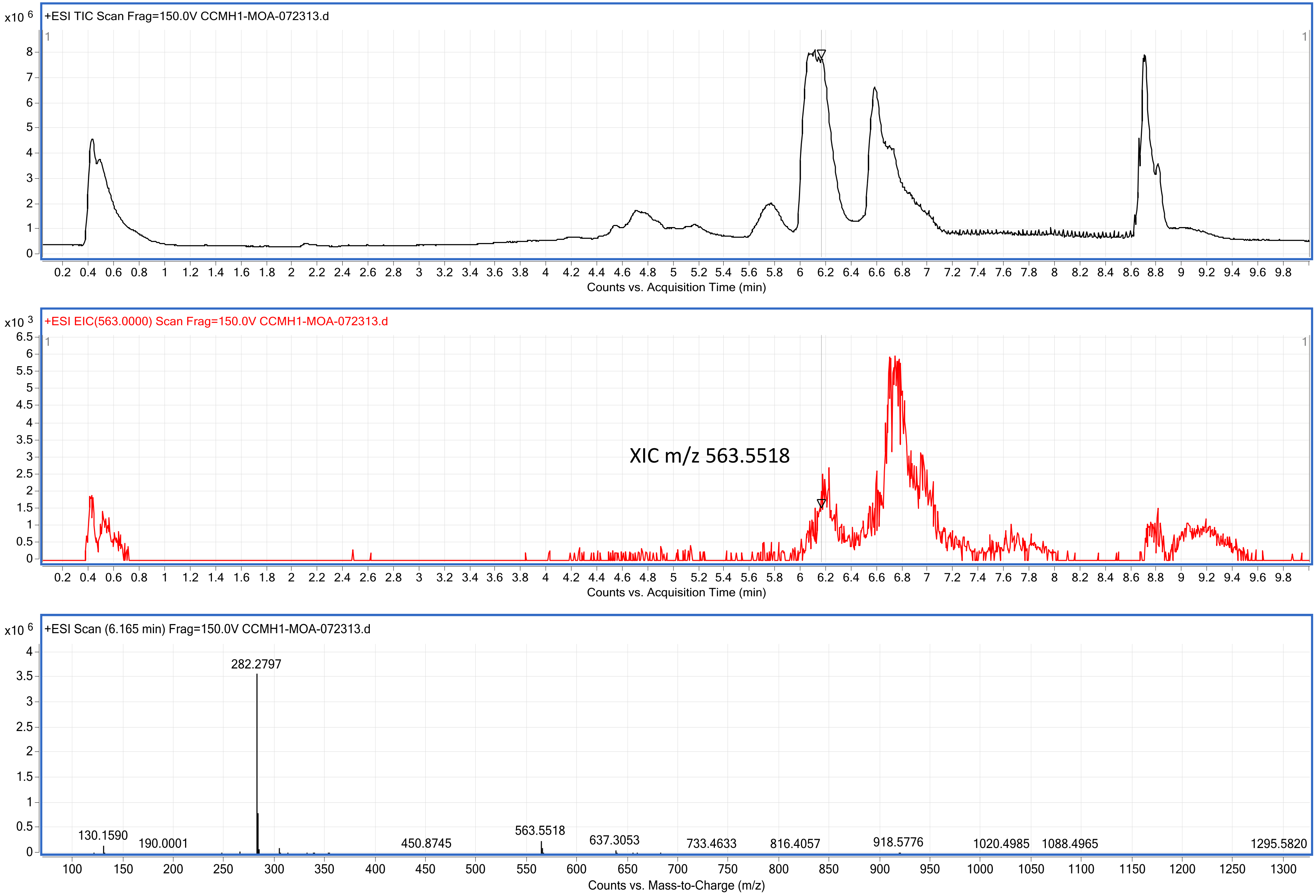

+ESI TIC Scan Frag=150.0V CCMH1-MOA-072313.d
Counts vs. Acquisition Time (min)
+ESI EIC(563.0000) Scan Frag=150.0V CCMH1-MOA-072313.d
XIC m/z 563.5518
Counts vs. Acquisition Time (min)
+ESI Scan (6.165 min) Frag=150.0V CCMH1-MOA-072313.d
282.2797
130.1590
190.0001
450.8745
563.5518
637.3053
733.4633
816.4057
918.5776
1020.4985
1088.4965
1295.5820
Counts vs. Mass-to-Charge (m/z)

Greater rhea (at 563)

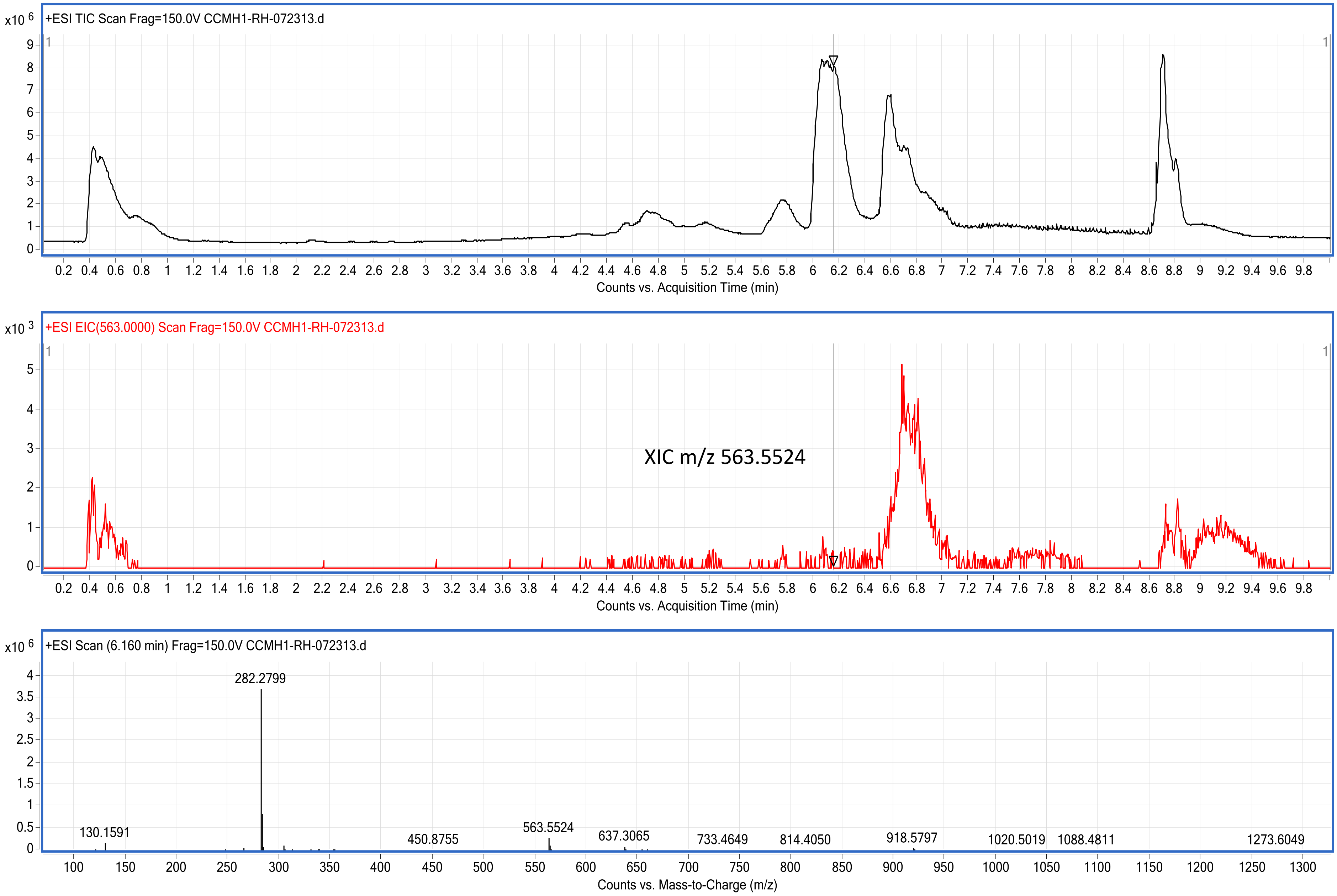

Chilean tinamou (at 563)

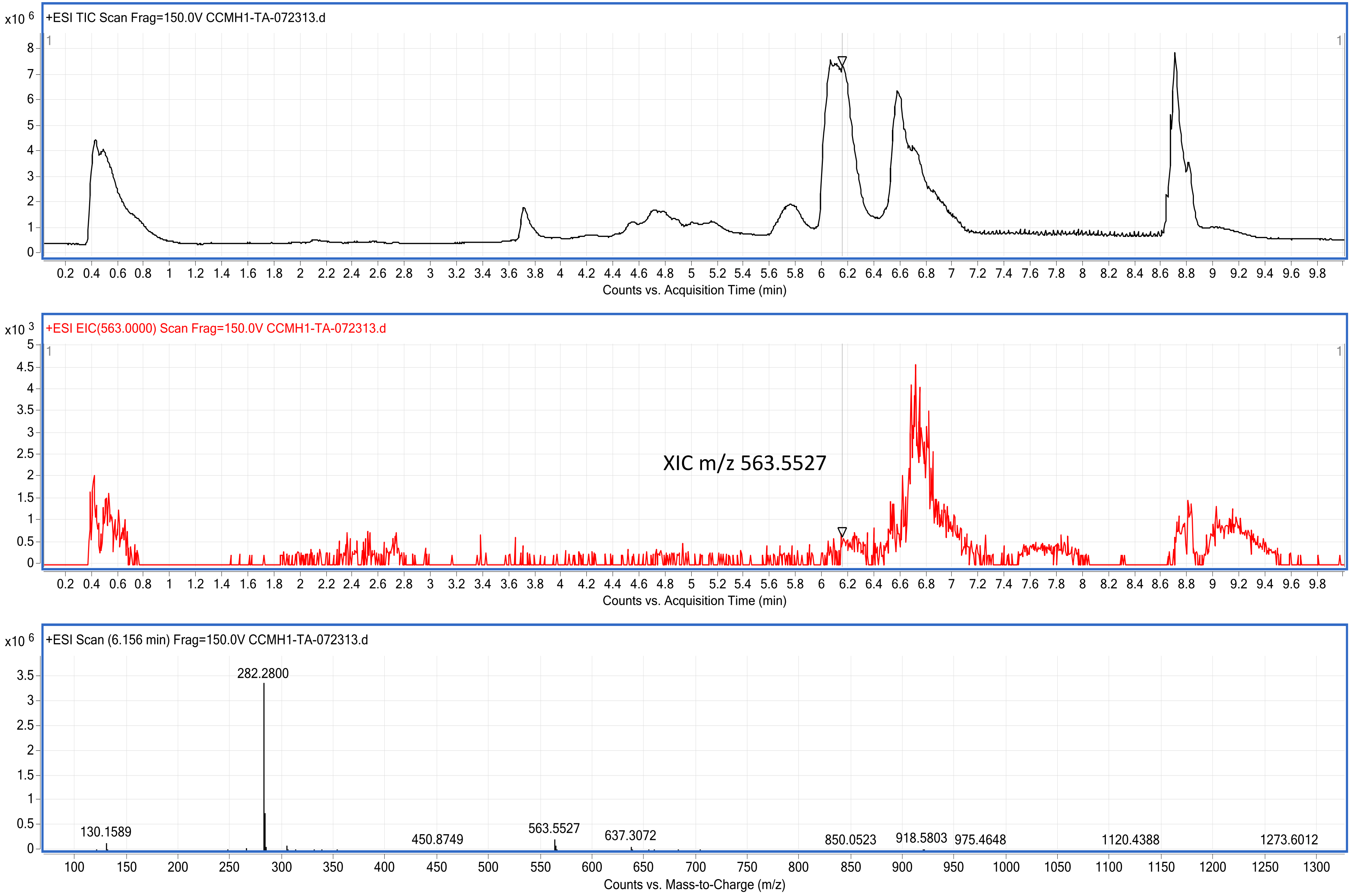

Chilean tinamou (at 583)

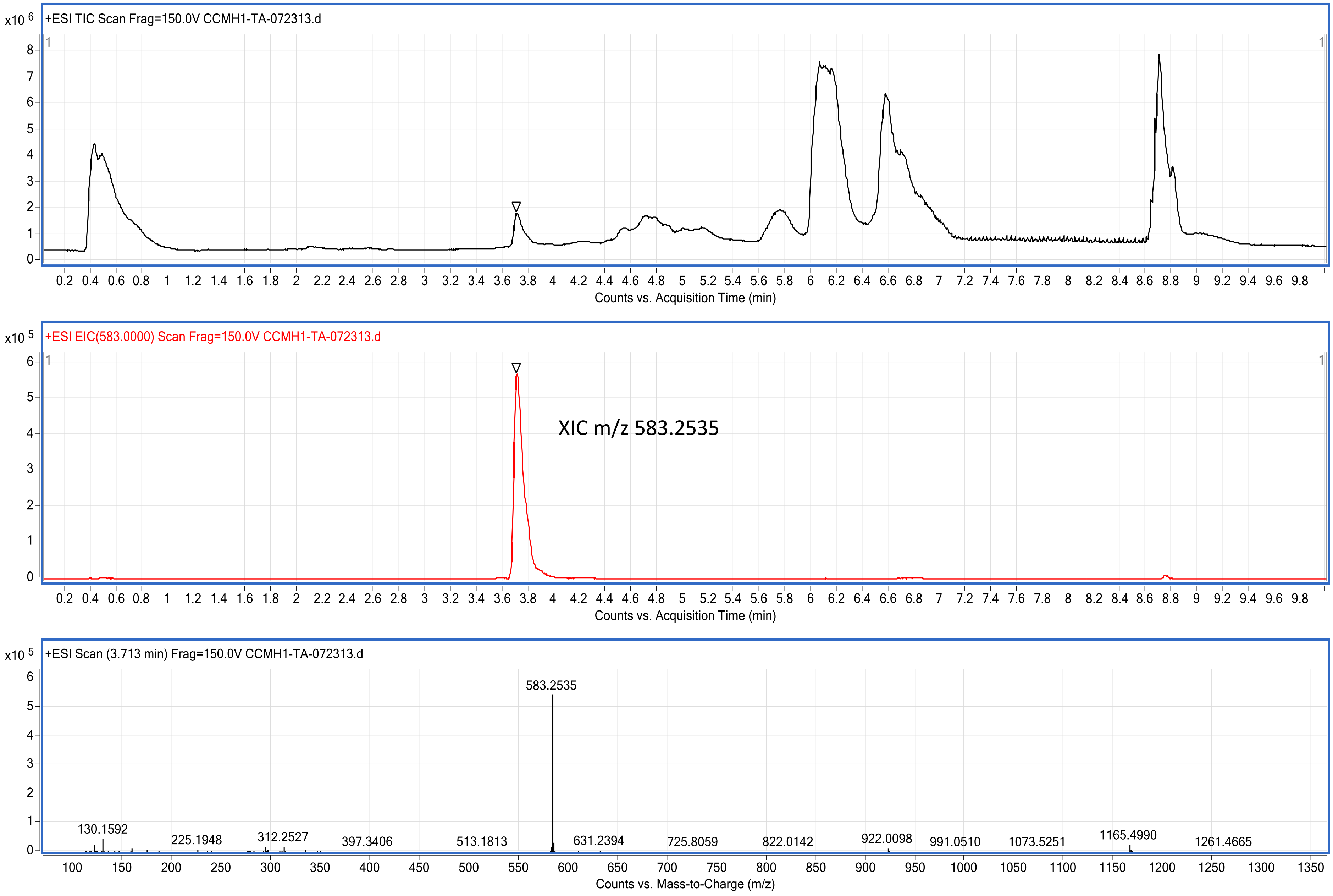

Great tinamou (at 563)

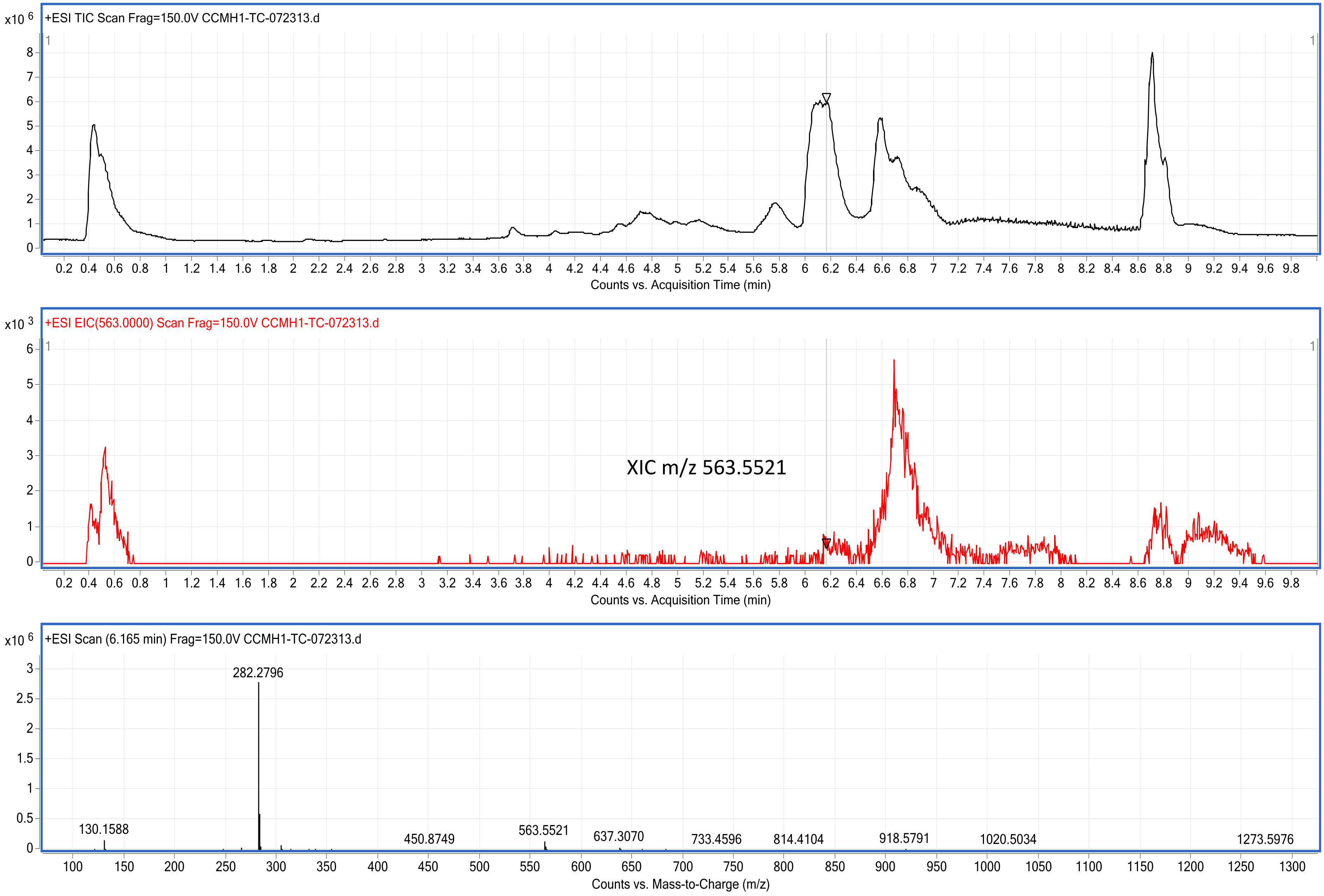

Great tinamou (at 583)

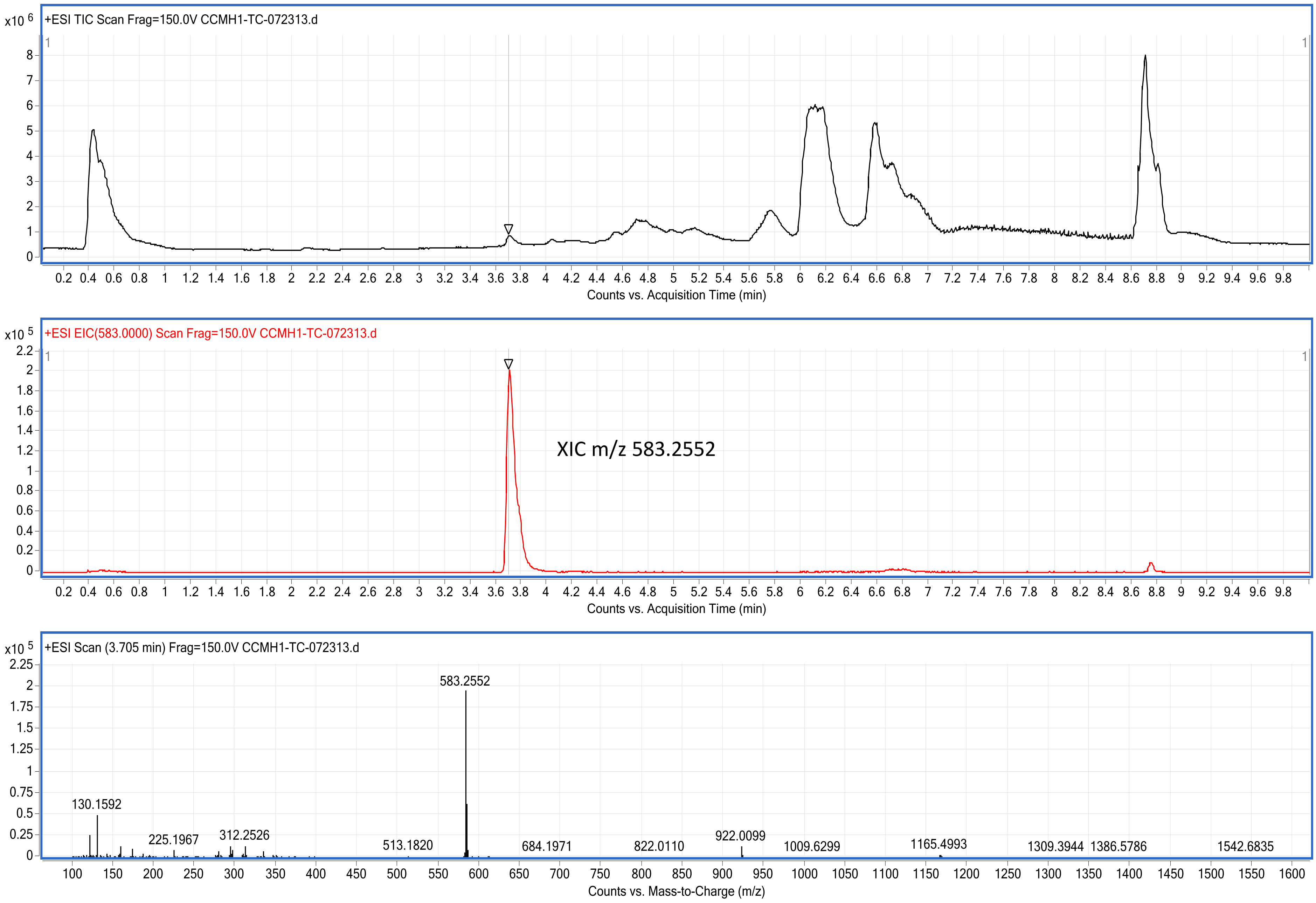

Spotted Nothura (at 563)

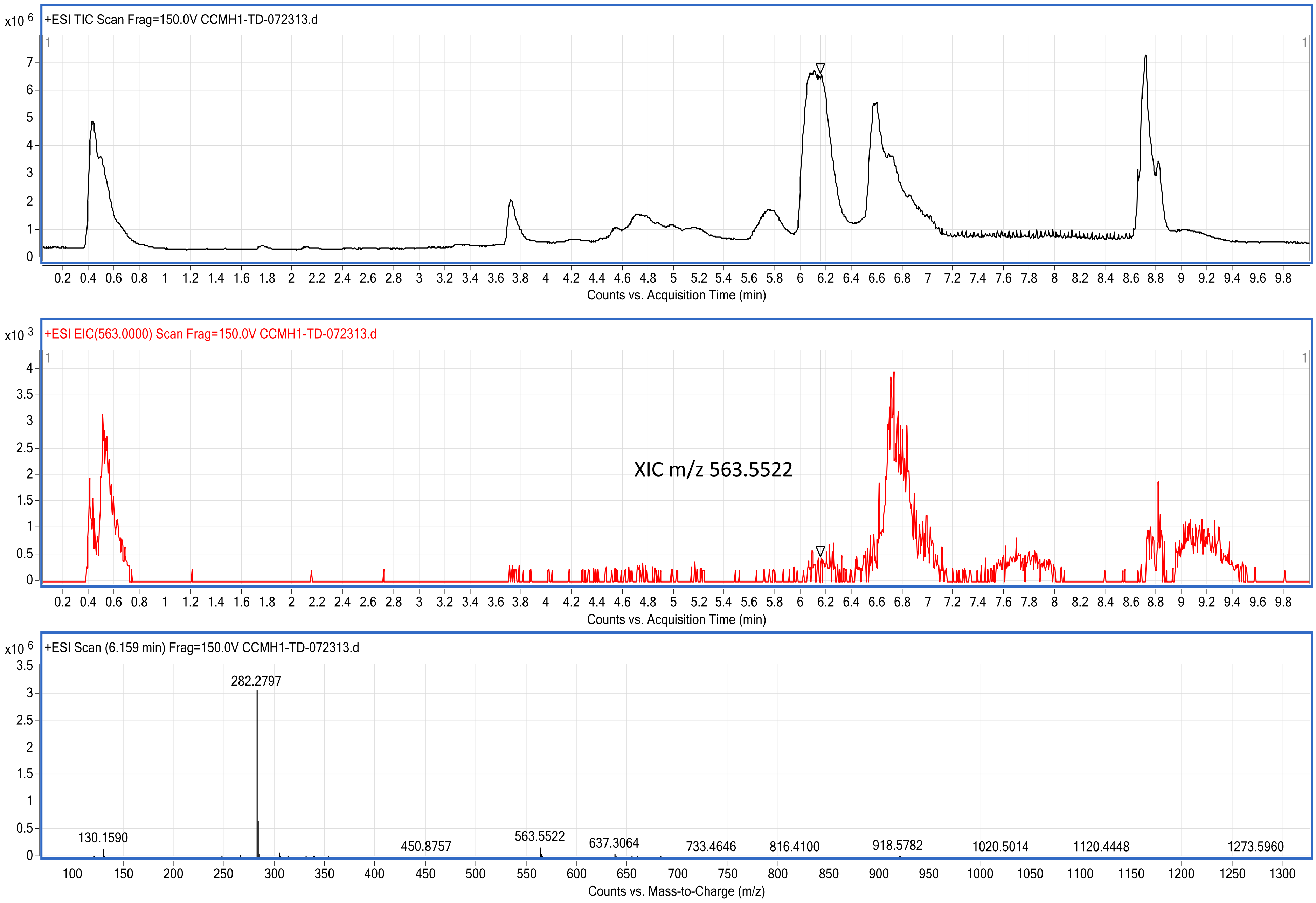

Spotted Nothura (at 583)

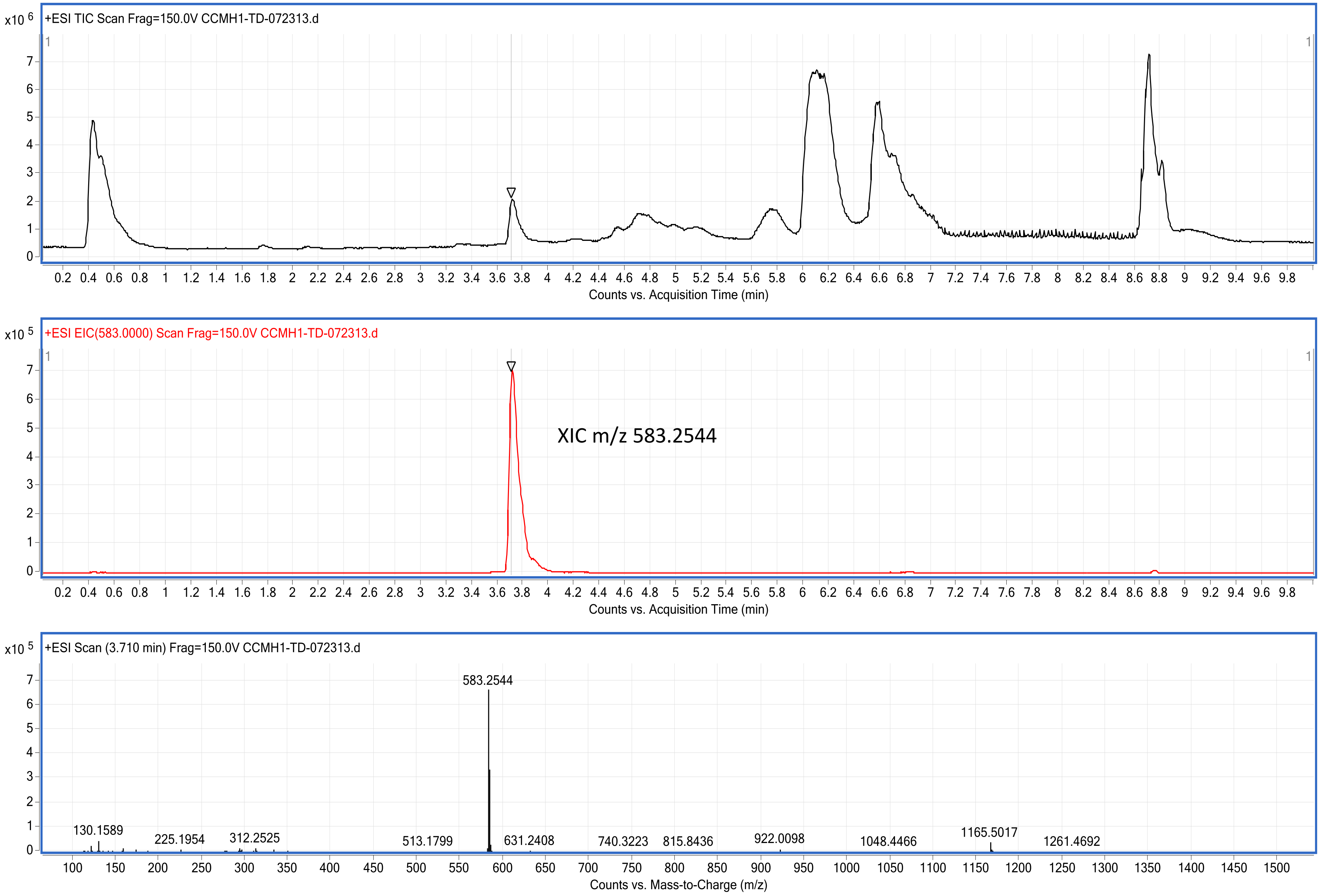

Elegant crested tinamou (at 583)

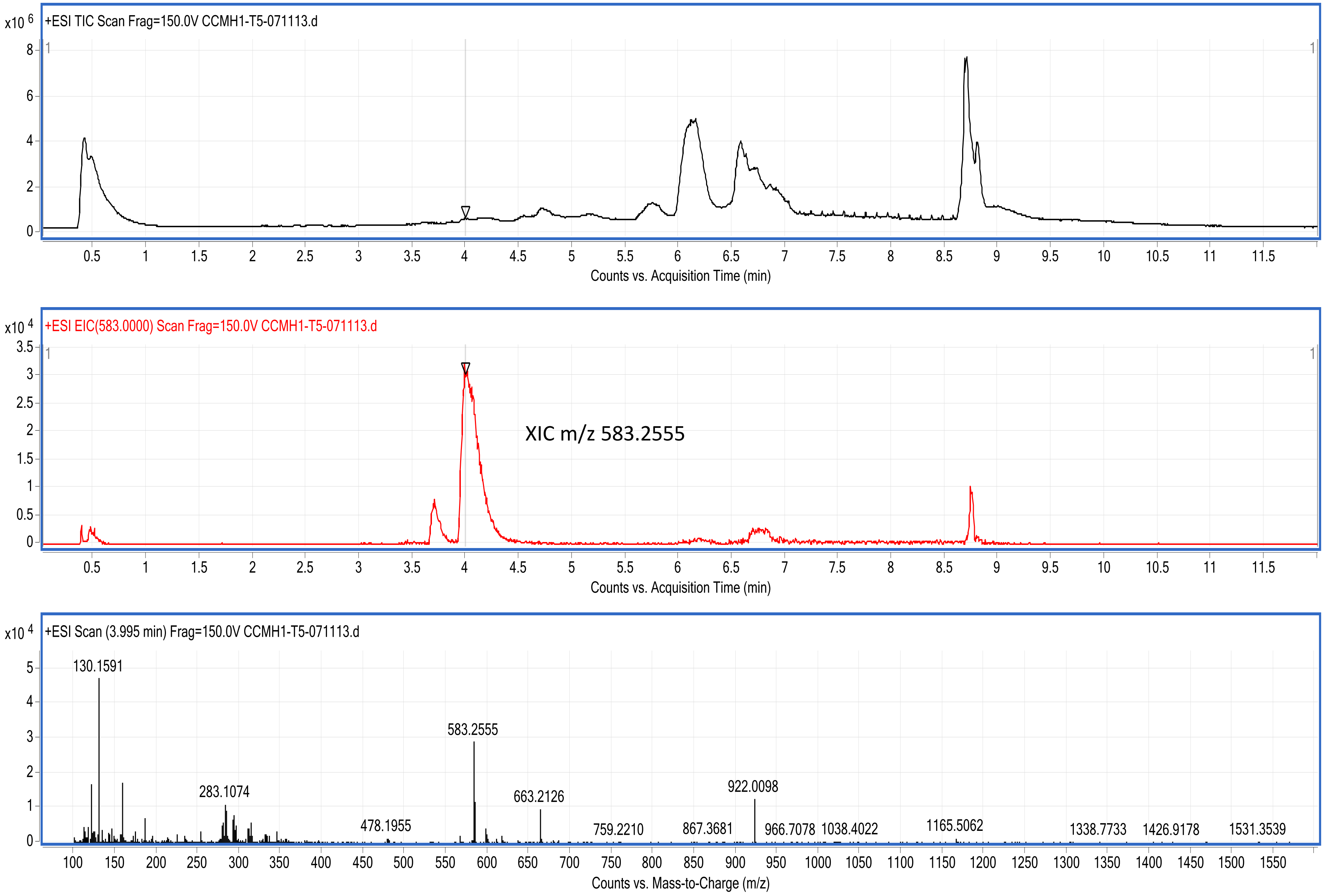

Bivalve sp. (at 563)

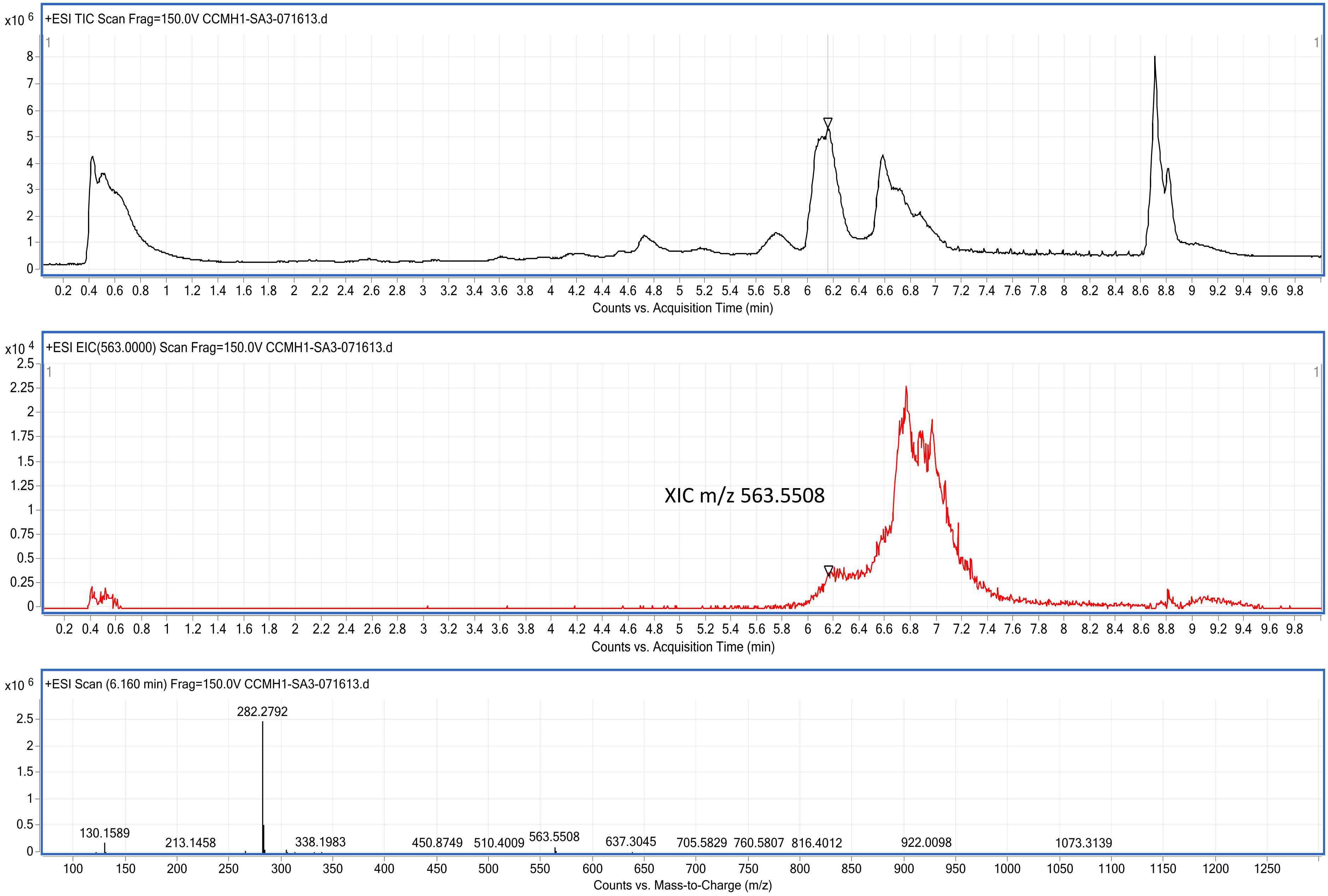

Conus striatus (at 563)

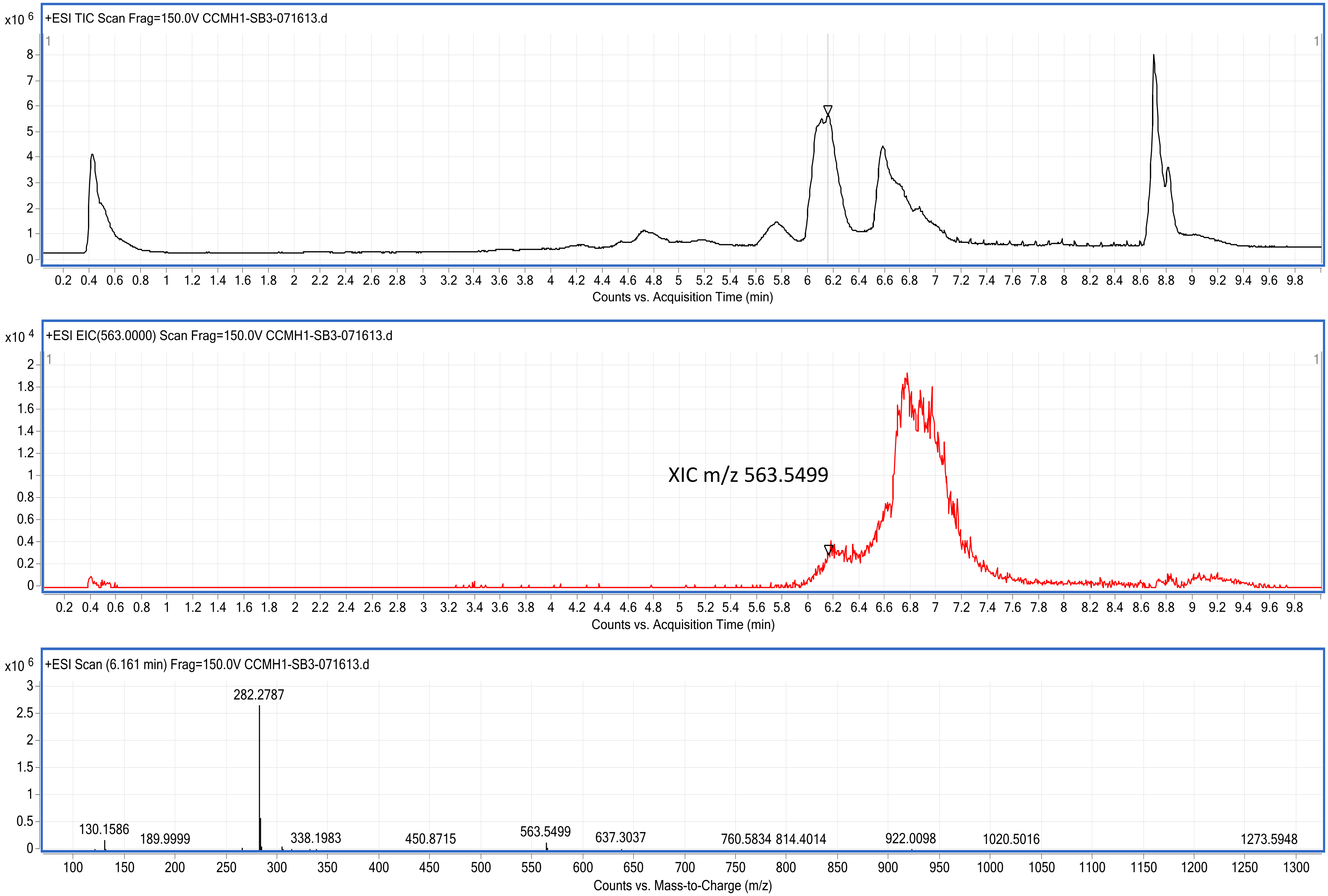

Hastula hectica (at 563)

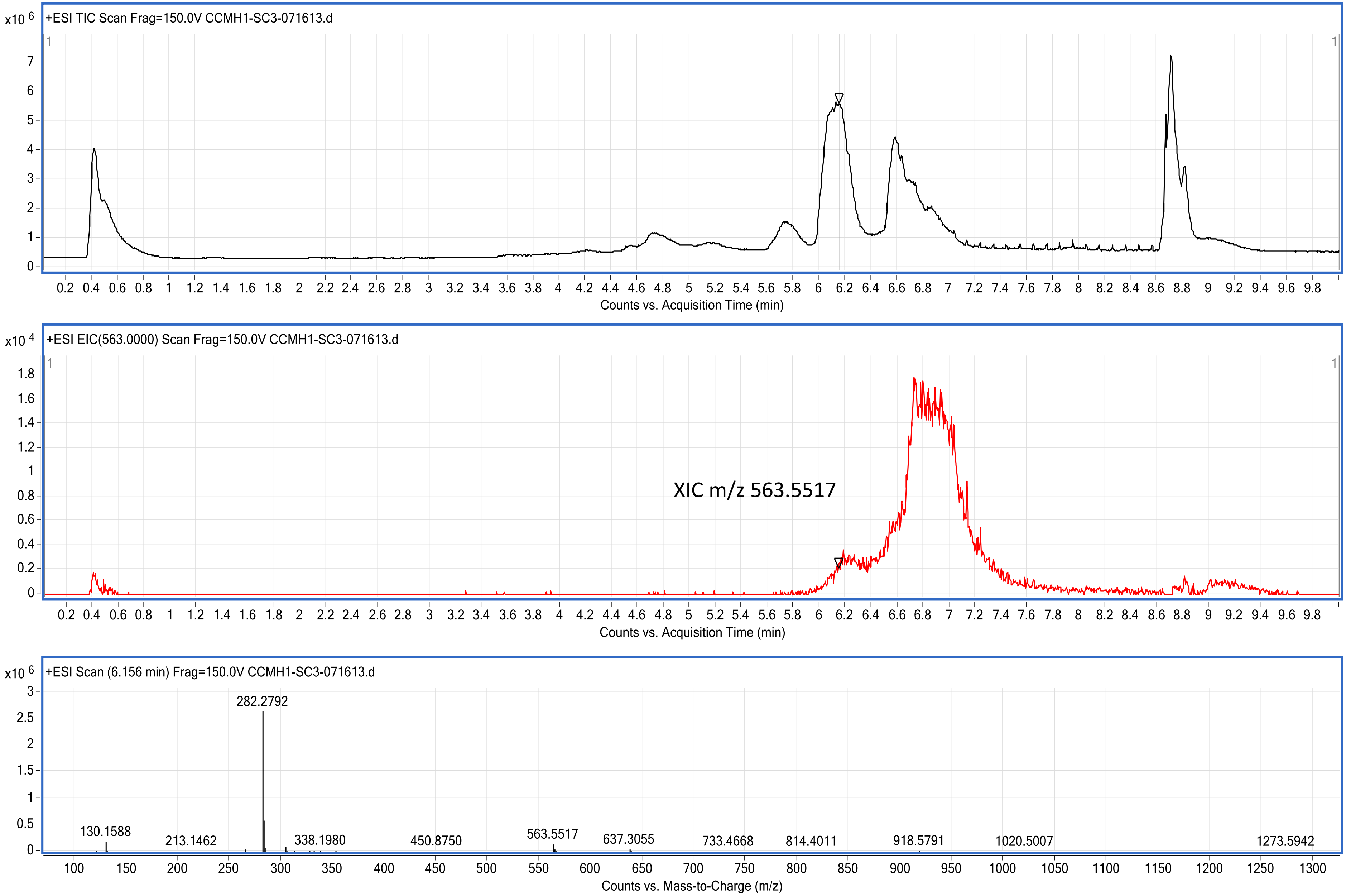

Conus ebraeus (at 563)

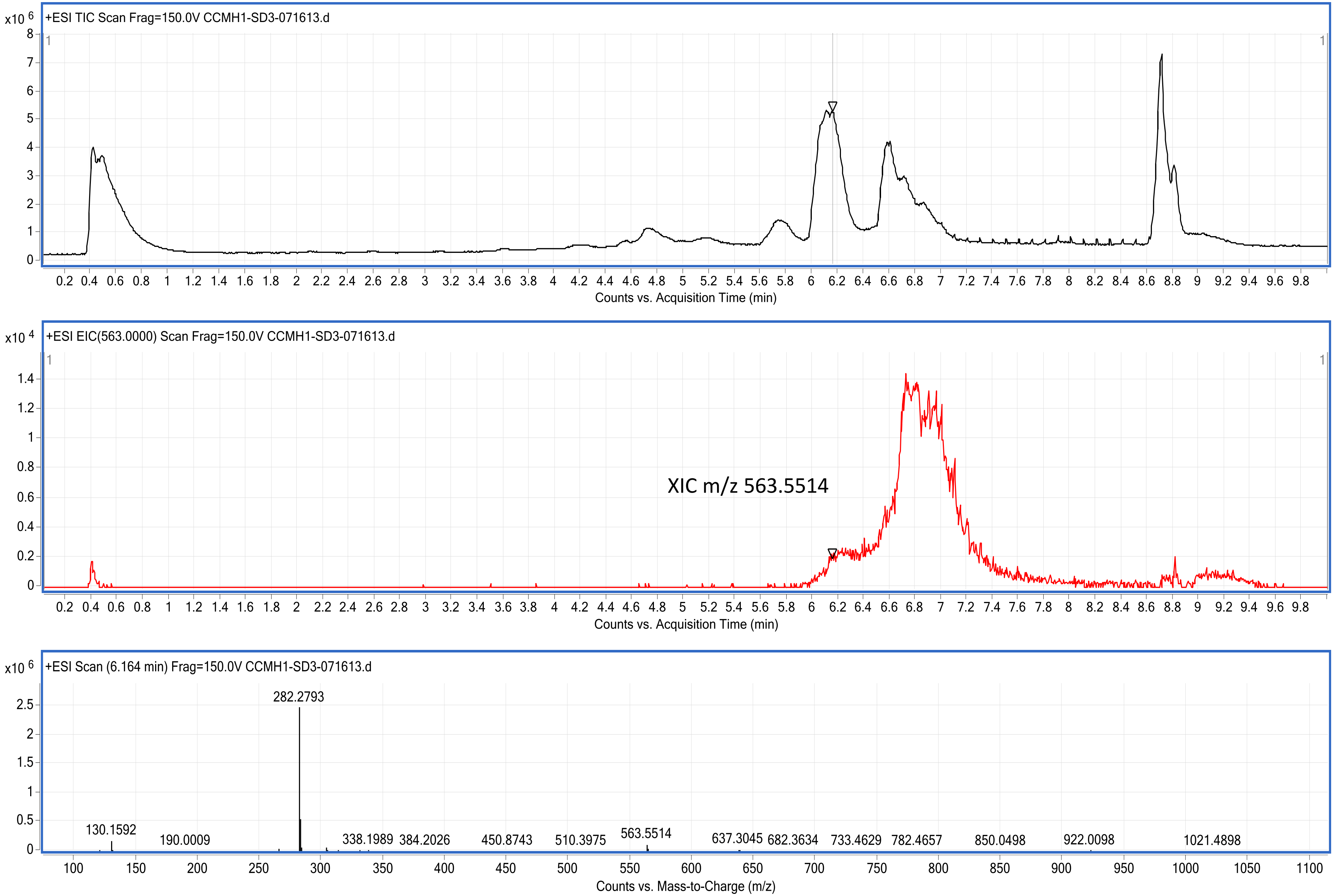

Protoporphyrin IX acid standard

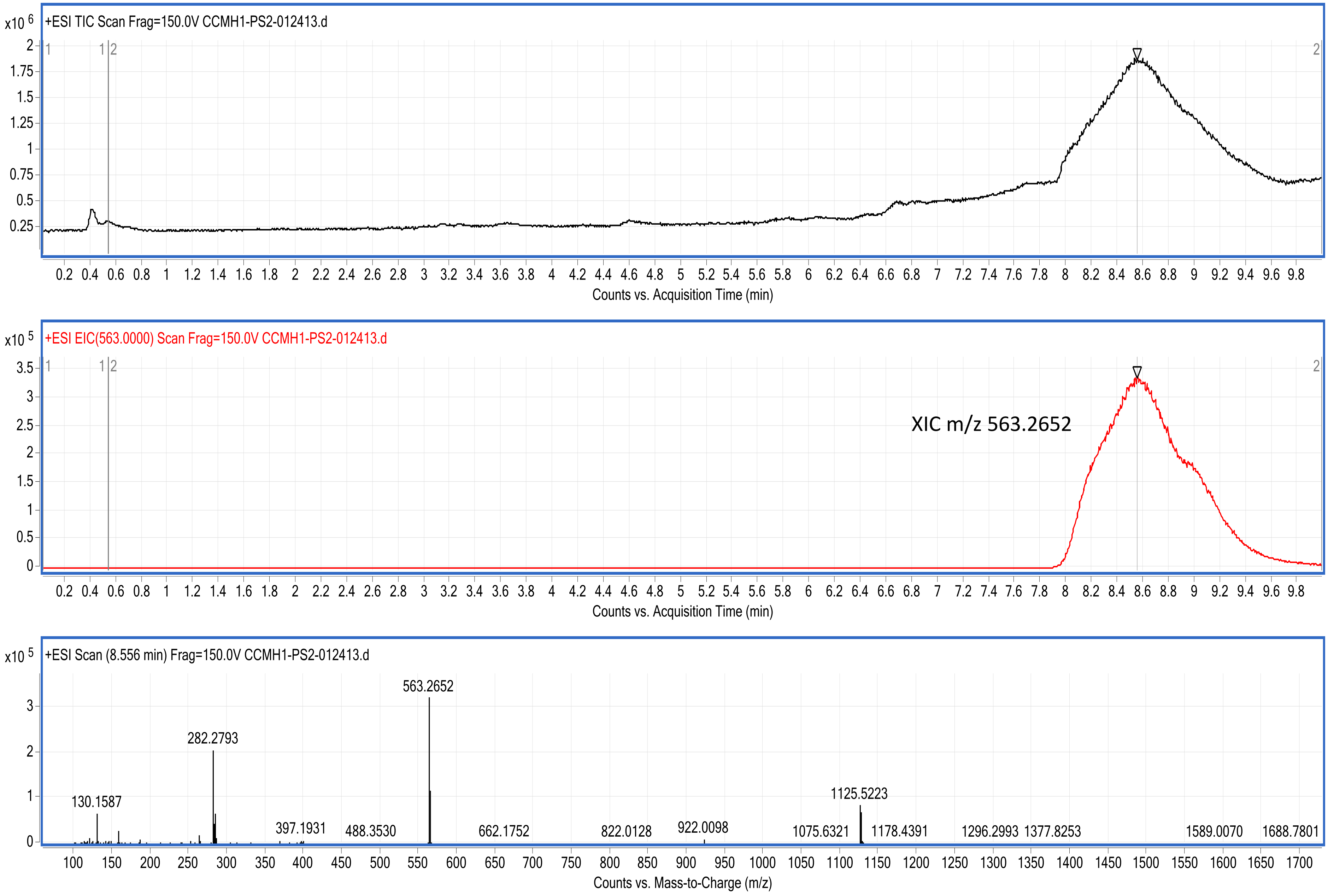

Supplement: S1 Fig — (PDF) [file pone.0143545.s001.pdf]
