# Supplementary material for: Nature’s Palette: Characterization of Shared Pigments in Colorful Avian and Mollusk Shells
Source: PLoS One. 2015 Dec 9;10(12):e0143545. doi: 10.1371/journal.pone.0143545 (PMC4674117; doi:10.1371/journal.pone.0143545)
Supplement: S1 Table — (PDF) [file pone.0143545.s002.pdf]

S2 Table. Shell purchase/collection locations.

Species	Location	Source
Euryapteryx curtus	Tokerau Beach, New Zealand	The Bone Room, Berkeley, CA, USA
Aepyornis sp.	Madagascar (extinct)	The Bone Room, Berkeley, CA, USA
Dromaius novaehollandiae	Akatarawa, New Zealand	Bluebank Blueberry and Emu Farm,
Casuarius casuarius	Dallas, TX, USA	The Eggery Place
Rhea americana	Dallas, TX, USA	The Eggery Place
Struthio camelus	Dallas, TX, USA	The Eggery Place
Apteryx mantelli	Rotorua, New Zealand	Rainbow Springs Kiwi House
Nothoprocta perdicaria	Dallas, TX, USA	The Eggery Place
Eudromia elegans	Bronx, NY, USA	Bronx Zoo
Tinamus major	La Selva, Costa Rica	Free-living bird's nest
Nothura maculosa	Dallas, TX, USA	The Eggery Place
Turdus migratorius	Ithaca, NY, USA	Free-living bird's nest
Molothrus ater	Ithaca, NY, USA	Free-living bird's nest
Coturnix japonica	Dallas, TX, USA	The Eggery Place
Gallus gallus	Dallas, TX, USA	The Eggery Place
Alligator mississippiensis	Dallas, TX, USA	The Eggery Place
Hastula hectica	Las Perlas Archipelago, Panama	Collection by SCUBA
Conus purpurascens	Las Perlas Archipelago, Panama	Collection by SCUBA
Conus ebraeus	Okinawa, Japan	Collection by SCUBA
Agropecten sp.	Las Perlas Archipelago, Panama	Collection by SCUBA
